# Normal stroma suppresses cancer cell proliferation via mechanosensitive regulation of JMJD1a-mediated transcription

**DOI:** 10.1038/ncomms12237

**Published:** 2016-08-04

**Authors:** Riina Kaukonen, Anja Mai, Maria Georgiadou, Markku Saari, Nicola De Franceschi, Timo Betz, Harri Sihto, Sami Ventelä, Laura Elo, Eija Jokitalo, Jukka Westermarck, Pirkko-Liisa Kellokumpu-Lehtinen, Heikki Joensuu, Reidar Grenman, Johanna Ivaska

**Affiliations:** 1 Centre for Biotechnology, University of Turku, 20520 Turku, Finland; 2 Institute of Cell Biology, Center for Molecular Biology of Inflammation, 48149 Muenster, Germany; 3 Laboratory of Molecular Oncology, Translational Cancer Biology Program, University of Helsinki, 00290 Helsinki, Finland; 4 Department of Otorhinolaryngology, Head and Neck Surgery, Turku University and Turku University Hospital, 20521 Turku, Finland; 5 Department of Mathematics and Statistics, University of Turku, 20520, Turku, Finland; 6 Institute of Biotechnology, Electron Microscopy Unit University of Helsinki, 00014 Helsinki, Finland; 7 Department of Oncology, Tampere University Hospital, 36280 Tampere, Finland; 8 Department of Oncology, Helsinki University Central Hospital, 00029 Helsinki, Finland; 9 Department of Biochemistry and Food Chemistry, University of Turku, 20520 Turku, Finland

## Abstract

Tissue homeostasis is dependent on the controlled localization of specific cell types and the correct composition of the extracellular stroma. While the role of the cancer stroma in tumour progression has been well characterized, the specific contribution of the matrix itself is unknown. Furthermore, the mechanisms enabling normal—not cancer—stroma to provide tumour-suppressive signals and act as an antitumorigenic barrier are poorly understood. Here we show that extracellular matrix (ECM) generated by normal fibroblasts (NFs) is softer than the CAF matrix, and its physical and structural features regulate cancer cell proliferation. We find that normal ECM triggers downregulation and nuclear exit of the histone demethylase JMJD1a resulting in the epigenetic growth restriction of carcinoma cells. Interestingly, JMJD1a positively regulates transcription of many target genes, including *YAP*/TAZ *(WWTR1)*, and therefore gene expression in a stiffness-dependent manner. Thus, normal stromal restricts cancer cell proliferation through JMJD1a-dependent modulation of gene expression.

## Abstract (web summary)

The tumour stroma has altered stiffness and matrix architecture compared to normal tissue, which favours proliferation, and invasion. Here, the authors find that the extracellular matrix produced by normal fibroblasts inhibits cancer cell proliferation through mechanosensitive downregulation of JMJD1a.

## Graphical abstract

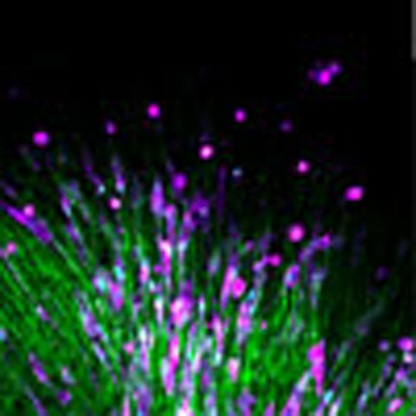

In normal tissue, different cell types are spatially confined. For example, the normal epithelium is separated from the underlying stromal extracellular matrix (ECM) by the basement membrane, and thus they are not in a direct contact with each other. The stromal ECM is produced by the fibroblasts and serves as an important regulator of tissue homeostasis^1^. In contrast, invasive carcinomas contain a complex mixture of tumour cells and stromal components where the cancer cells interact with the altered ECM and are also embedded in it. Changes in the matrix stiffness as well as transition between two-dimensional and three-dimensional matrix contacts influence cell proliferation profoundly^2^,^3^,^4^,^5^. Thus, cancer progression involves not only genetic alterations of cancer cells but also changes in the tumour microenvironment^6^. The role of the stroma as a potent antitumorigenic barrier has already been elucidated over two decades ago^7^,^8^. The exposure of carcinoma cells to normal basement membrane-like components has the capacity to revert breast cancer cells to a near-normal phenotype^9^. Furthermore, in coculture systems, normal fibroblasts (NFs) can inhibit the growth of certain cancer cells^10^,^11^, while a population of transformed cancer-associated fibroblasts (CAFs) can reverse the growth-inhibiting effect of NFs^12^. Thus, cross-talk between epithelial cells and fibroblasts is a critical feature of cancer progression. However, the specific contribution of the matrix itself has not been addressed in detail.

Tumour stromata are characterized by increased tissue stiffness and altered matrix architecture that favour proliferation, metastasis and drug resistance via aberrant mechanosignalling^13^,^14^,^15^. Stiffness also activates the main mediators of mechanotransduction: transcription factors YAP (Yes-associated protein) and TAZ (transcriptional coactivator with PDZ-binding motif)^16^. Epigenetic regulation is also implicated in cancer progression as it profoundly regulates the transcription profile and phenotype of cells. Many lines of evidence suggest that demethylation of repressive histone methylation marks, such as histone H3 lysine 9 (H3K9), from the gene promoters by histone demethylases is a prerequisite for transcriptional activation^17^,^18^,^19^. JMJD1A (KDM3A) demethylates monomethyl and dimethyl histone H3K9 *in vitro* and *in vivo* and has been implicated as a positive regulator of transcription of several growth-promoting genes^17^,^18^,^19^.

While the mechanisms whereby cancer stroma and CAFs contribute to tumour progression are being actively investigated, much less is known about how the normal stroma exerts tumour-suppressive signals to control tissue homeostasis. Furthermore, the role of epigenetic regulators in the ability of the cells to respond to the stiffness of the tumour microenvironment is not known. To investigate this, we compared the ability of matrices generated by NFs or CAFs from the same patient as well as matrices from immortalized NFs to influence cancer cell proliferation and gene expression. We find that the matrix generated by NFs, but not CAF matrix, profoundly inhibits cancer cell proliferation through mechanosensitive downregulation of the histone demethylase enzyme JMJD1a.

## Results

### Normal matrix inhibits cancer cell proliferation

**Figure 1:**
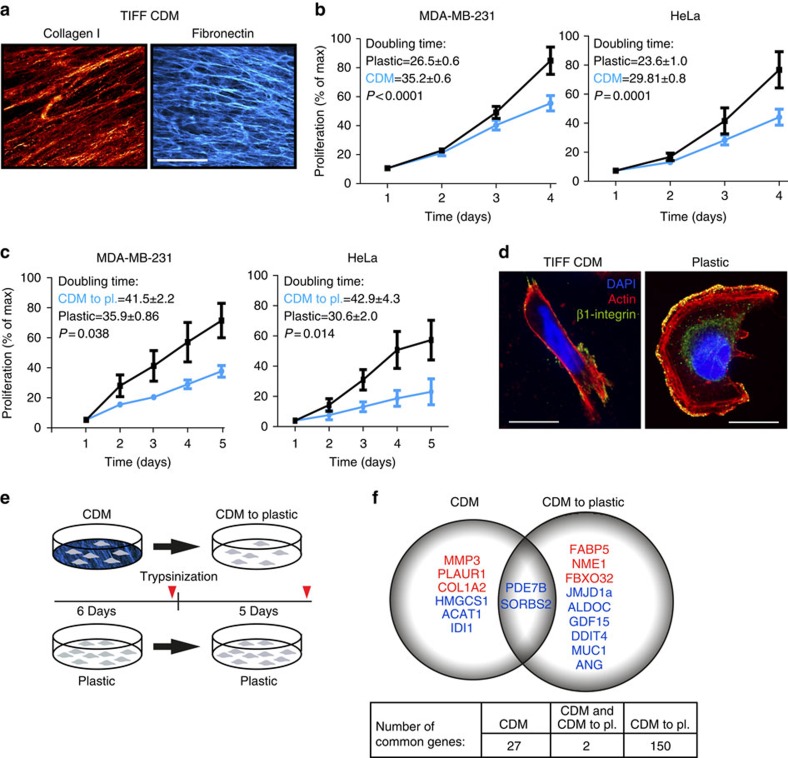
Fibroblast-derived CDM induces sustained growth inhibition of cancer cells. (**a**) Collagen I and fibronectin staining of CDM generated by NFs (TIFFs). Scale bar, 20 μm. (**b**) Proliferation of MDA-MB-231 and HeLa cells plated on TIFF CDM or on plastic in full medium for the indicated times. *n*(HeLa)=10, *n*(MDA-MB-231)=8. (**c**) Proliferation of MDA-MB-231 and HeLa cells after detachment from TIFF matrices (6 days on matrix before detachment) and replating on plastic in full medium for the indicated times *n*(HeLa)=7, *n*(MDA-MB-231)=10. (**d**) Representative images of MDA-MB-231 cell morphology on CDM and plastic. Shown are maximum intensity projections of confocal images. Scale bar, 10 μm. (**e**) Schematic representation of the experimental set-up. Red arrows indicate the time points of sample collection for Illumina gene expression analysis. (**f**) Common gene expression changes in MDA-MB-231 and HeLa cells on CDM and 5 days after CDM detachment (CDM to plastic (pl.)). The numbers of commonly regulated genes (up- or downregulated) in both cell lines are indicated in the table. Upregulated genes are marked with red and downregulated genes with blue. All data are mean±s.e.m. Unpaired *t*-test was used for statistical analyses.

Fibroblasts produce *in vitro* a robust cell-derived matrix (CDM) that recapitulates many features of the architecture and composition of *in vivo* ECM^20^. To investigate the potential effect of matrix on cancer cell proliferation, we generated matrices from telomerase-immortalized NFs (TIFFs) (Fig. 1a) and tested their effectiveness on two highly proliferative and widely studied cancer cell lines, namely cervical cancer HeLa and breast cancer MDA-MB-231 cells. Remarkably, both of these cell lines were significantly growth-inhibited (Fig. 1b) by the normal matrix compared with standard growth conditions on plastic. The growth-restrictive properties of CDM were only observed with the intact CDM as matrix proteins such as fibronectin or collagen I, or solubilized and re-plated CDM did not inhibit MDA-MB-231 cell proliferation (Supplementary Fig. 1a,b). Soluble factors were not implicated either because culturing the MDA-MB-231 cells in conditioned TIFF medium did not influence proliferation (Supplementary Fig. 1c). Thus, only the architecturally intact CDM possessed growth-inhibitory properties.

When investigating matrix-induced effects in more detail we, rather unexpectedly, observed that growth inhibition induced by TIFF CDM was maintained in cancer cells following detachment from the matrix by trypsinization and replating on plastic (Fig. 1c). Even though the matrix-exposed cells were returned to plastic in full serum-containing medium, both MDA-MB-231 and HeLa cells continued to proliferate significantly slower than the same cancer cell lines cultured continuously on plastic. Thus, exposure of cancer cells to CDM from NFs is not only growth-inhibitory but has the potential to revert malignant cancer cell proliferation in a sustained manner.

We observed that cancer cells grown on normal TIFF-derived CDM had significantly altered cell morphology compared with cells on plastic (Fig. 1d and Supplementary Fig. 1d). This was interesting as mechanical cues and environmental stiffness are known to affect the cytoskeleton and nuclear functions including chromatin condensation and global epigenetic status of a cell^21^,^22^,^23^,^24^,^25^, and therefore changes in cell morphology and gene expression could explain the matrix-dependent reversion in the cancer cell phenotype.

### Matrix induces gene expression changes

We hypothesized that exposure to CDM could induce changes in epigenetic modifiers, hence suppressing cancer cell growth in a sustained manner. To investigate this possibility, we performed Illumina whole-genome transcription analysis in MDA-MB-231 and HeLa cells harvested directly from TIFF CDMs after 6 days (CDM), following detachment from CDMs and replating on plastic for 5 days (CDM to plastic) or in cells grown continuously on plastic (Fig. 1e). As CDM induced sustained growth inhibition in both cell lines, we focused our attention on common transcriptional alterations of epigenetic enzymes. A single well-characterized histone demethylase JMJD1a was significantly downregulated in both cell lines, suggesting that this gene might be linked to the sustained phenotypic alterations triggered by the CDM in both cancer cell lines. Among the other significantly altered genes were signalling proteins *SORBS2* and *PDE7B*, which were downregulated in both cell lines and in both conditions (CDM and CDM to plastic; Fig. 1f and Supplementary Data 1). In addition, expression of 27 genes on CDM and 150 genes following CDM detachment were altered significantly in both cell lines. Several matrix-modifying proteins (MMP3, PLAUR1 and COL1A2) were upregulated on the matrix in both cell lines, while many genes involved in regulating cholesterol synthesis (*IDI1*, *ACAT1* and *HMGCS1*) were downregulated on the matrix. Following matrix detachment, several hypoxia-related genes were downregulated (JMJD1A *(KDM3A)*, *ALDOC*, *DDIT4*, *GDF15*, *ANG* and *MUC1*) even though the rather thin CDM layer is unlikely to restrict oxygen diffusion in the *in vitro* cultures^26^. In addition, MYC-target genes (*FAPB5* and *NME1*) were upregulated following CDM detachment and replating on plastic. Since the mechanism of sustained growth inhibition was of primary interest, we focused specifically on the epigenetic modifier enzyme JMJD1a that was downregulated following matrix detachment in both cell lines (Fig. 1f).

### Normal matrix restrains JMJD1a expression

**Figure 2:**
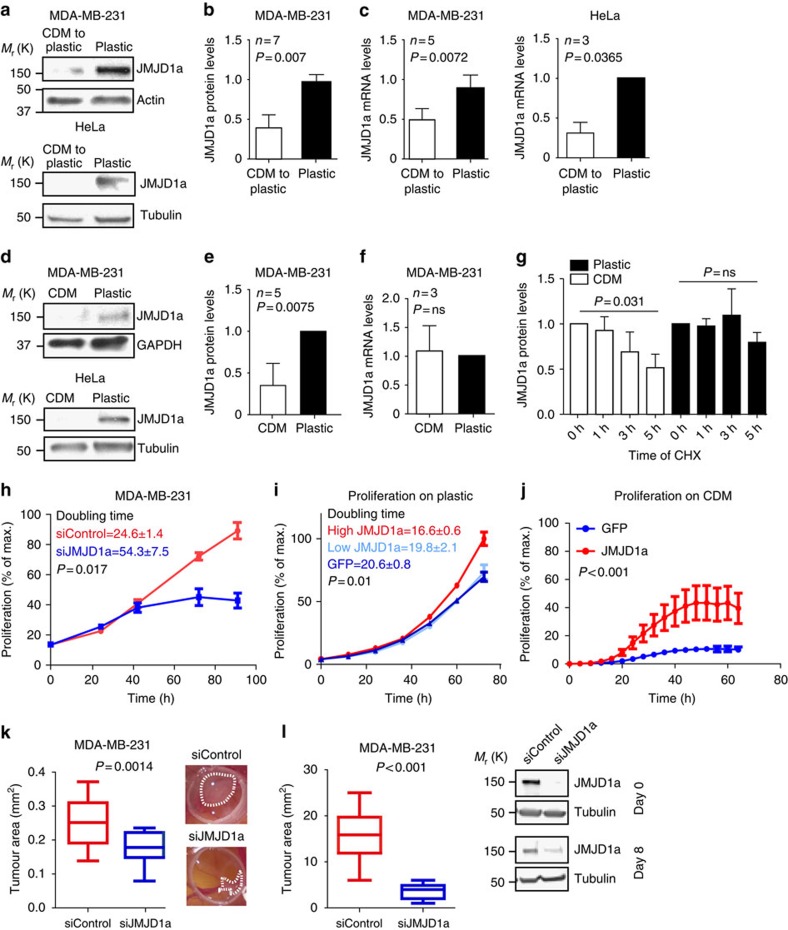
Normal ECM restrains JMJD1a expression, cell proliferation and tumour growth. (**a**,**b**) Western blot (**a**) and quantification (**b**) of JMJD1a protein levels normalized to loading control. (**c**) JMJD1a mRNA expression (qRT–PCR) relative to GAPDH mRNA in MDA-MB-231 and HeLa cells after matrix detachment (6 days on TIFF CDM and 5 days on plastic; CDM to plastic) and on plastic. (**d**,**e**) Western blot (**d**) and quantification of JMJD1a protein levels (**e**) normalized to loading control. (**f**) JMJD1a mRNA expression (qRT–PCR) relative to GAPDH mRNA (**f**) in the indicated cells plated on either TIFF CDM or on plastic for 4 days. (**g**) Western blot quantification showing JMJD1a stability on CDM and plastic 24 h after plating. Time of the cycloheximide (CHX) treatment is indicated and *P* values are calculated between 0 and 5 h. Paired *t*-test was used for statistical analysis, *n*=3. (**h**) Proliferation of MDA-MB-231 cells upon JMJD1a silencing on plastic, *n*=3. (**i**) Proliferation of JMJD1a-GFP or GFP-overexpressing MDA-MB-231 cells on plastic. Cells were sorted by FACS (JMJD1a: high and low; GFP: high), *n*=3. (**j**) Proliferation of GFP control and JMJD1a-GFP-overexpressing MDA-MB-231 cells on TIFF-derived CDM. *n* (GFP)=11 CDMs and *n*(JMJD1a-GFP)=12 CDMs. Two-way analysis of variance (ANOVA) was used to calculate the *P* value. Data are mean±s.e.m. (**k**) Control or JMJD1a siRNA-transfected MDA-MB-231 cells (1 × 10^6^) were implanted on CAM membranes inside a plastic ring to analyse tumour growth *in vivo* for 3 days. Shown are quantified tumour areas from three individual experiments *n*(siControl)=25, *n*(siJMJD1a)=23 eggs. (**l**) Orthotopic tumour growth assay. Control or JMJD1a siRNA-transfected MDA-MB-231 cells (2 × 10^6^) were injected into the fat pad of nude mice (*n*=19) and tumour growth was measured 8 days after injection. Western blot in showing the silencing efficacy of JMJD1a siRNA on the day of the injection (Day 0) and at the end of the experiment (Day 8). Shown are mean±s.d. and (**g**–**i**) mean±s.e.m. Paired *t*-test was used for statistical analyses in **b**–**h** and non-paired *t*-test in **j**,**k**.

JMJD1a is a H3K9-specific demethylase that has been linked to several biological processes including growth, development and reprogramming^17^,^27^,^28^,^29^. Loss of JMJD1a decreases gene expression and increases the inhibitory H3K9me2 modifications in the promoter regions of multiple genes^19^. We validated that JMJD1a was downregulated in cancer cells following detachment from CDM both on the protein (Fig. 2a,b) and on mRNA levels (Fig. 2c). Interestingly, JMJD1A was downregulated already in cells grown on CDM at the protein level (Fig. 2d,e), while the mRNA levels of JMJD1a were not changed (Fig. 2f). This correlated with reduced stability of the JMJD1a protein on CDM when compared with plastic (Fig. 2g), indicating that CDM-induced repression of JMJD1a first on the protein level and subsequently on mRNA level correlates with the CDM-induced growth inhibition of cancer cells when compared with cells grown on plastic (Fig. 1b,c). Silencing of JMJD1a recapitulated the growth inhibition in plastic-cultured cancer cells and induced a flat quiescent-looking morphology (Fig. 2h and Supplementary Fig. 1e–f). Conversely, proliferation was enhanced in cells with high JMJD1a-GFP levels when compared with high green fluorescent protein (GFP)-expressing cells on plastic (Fig. 2i and Supplementary Fig. 1g). Importantly, forced expression of JMJD1a was sufficient to rescue the CDM-induced growth inhibition on TIFF-derived CDM (Fig. 2j). Furthermore, the growth of MDA-MB-231 (breast cancer) cells in an *in vivo* chicken embryo chorioallantoic membrane (CAM) assay as well as orthotopic tumour growth in mice were significantly reduced upon silencing of JMJD1a (Fig. 2k,l). Thus, JMJD1a is downregulated following CDM exposure of cells and is a potent regulator of cancer cell proliferation *in vitro* and *in vivo*.

### NFs and CAFs generate architecturally distinct CDM

**Figure 3:**
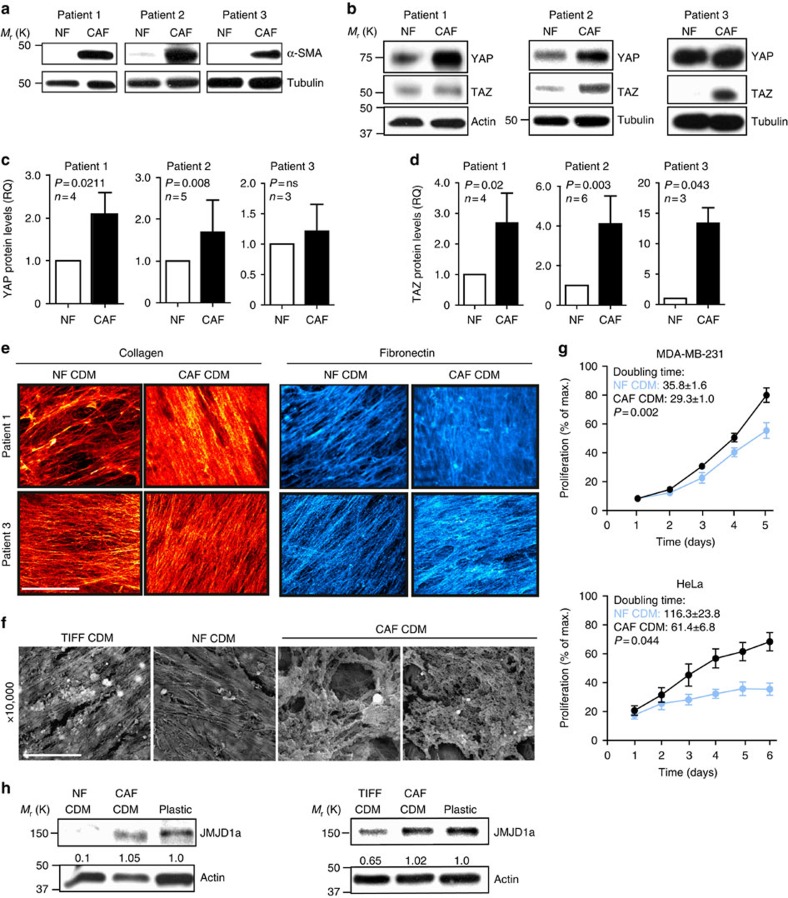
Patient-derived CAF and NF CDMs are architecturally and functionally distinct. (**a**,**b**) Representative western blots showing SMA-α (**a**) and YAP/TAZ expression (**b**) in NFs and CAFs isolated from three SCC patients. (**c,d**) Quantification of YAP (**c**) and TAZ (**d**) expression in NFs and CAFs normalized to loading control. Data are mean±s.d. (**e**) Collagen I (red) and fibronectin (blue) staining of patient #1 and #3 NF and CAF CDM. Scale bar, 20 μm. (**f**) Representative SEM images of TIFF, Patient #1 NF and CAF CDM. Scale bar, 5 μm. (**g**) Proliferation of MDA-MB-231 and HeLa cells on Patient #1 NF and CAF CDM. *n*(MDA-MB-231)=10–11 and *n*(HeLa)=19 from three independent experiments. Data are mean±s.e.m. and *P* values are calculated from the doubling times. (**h**) JMJD1a expression in MDA-MB-231 cells cultured on TIFF, NF and CAF CDMs and on plastic. Quantification shows relative JMJD1a amount normalized to loading control. (**c,d**) Non-parametric Mann–Whitney test was used for statistical analyses in **c,d** and non-paired *t*-test in **g**.

To test the ability of stromal ECM to influence cancer cells in a more clinically relevant model, we isolated CAFs from the tumour stroma of three head and neck squamous cell carcinoma (HNSCC) patients (Supplementary Fig. 2a) and NFs from an unaffected area of the same patients. CAFs were identified by the high expression of CAF marker smooth muscle actin-α (α-SMA), which was low or absent in the NFs and the TIFFs (Fig. 3a and Supplementary Fig. 2b). RNA-sequencing demonstrated that all three CAFs and NFs clustered together (Supplementary Fig. 2c,d), which indicated that CAFs derived from different patients were more similar to other CAFs than to their corresponding NFs and all the NFs resembled each other (Supplementary Fig. 2c,d). However, all NF and CAF cell lines expressed genes, which are typically highly expressed by fibroblasts, such as *Vimentin*, *Fibronectin 1* and several different collagens validating that they are fibroblasts (Supplementary Fig. 2e). CAFs had elevated levels of YAP and TAZ (Fig. 3b–d) compared with NFs, and they were also more able to contract collagen gels (Supplementary Fig. 2f), in line with a previous report on the role of YAP and contractility in the CAF phenotype^30^. In addition, we observed a significant upregulation of β1-integrin, which has also been connected to contractility and mechanosignalling (Supplementary Fig. 2g,h).

We analysed the CDM produced by the different fibroblasts using immunofluorescence and scanning electron microscopy (SEM). Immunofluorescence staining revealed that similarly to TIFF CDM (Fig. 1a) the matrix bundles in NF CDM were more sparse compared with the corresponding CAF matrix, which displayed a denser and more uniform collagen and fibronectin staining (Fig. 3e and Supplementary Fig. 3a,b). Furthermore, based on SEM analysis, the NF and TIFF CDMs had more uniform and aligned structures compared with CAF-derived matrices (Fig. 3f). According to the RNA-sequencing data, the mRNA expression of different types of collagen or fibronectin was not changed, suggesting that matrix assembly and/or turnover, rather than production, results in distinct CDM architecture between NF and CAF CDM. However, even though we could not find significant differences on mRNA levels of matrix-related genes or in cell adhesion to solubilized and re-plated NF and CAF CDM (Supplementary Fig. 3c), the NF and CAF CDMs may differ in their protein composition as suggested earlier by others^31^.

These data show that CDMs generated by NFs and CAFs differ and that CAF-derived matrices from different patients share similar features that are distinct from matrices made by NFs from the same individual.

### CAF matrix lacks growth-inhibitory properties

To test the ability of the patient-derived stromal ECM to influence the proliferation of cancer cells, we cultured MDA-MB-231 and HeLa cells on CDMs derived from either NFs or CAFs. Interestingly, NF CDM was significantly growth-inhibitory compared with CDM generated by CAFs from the same patient (Fig. 3g), and the same was observed when comparing TIFF and CAF CDM (Supplementary Fig. 3d). Importantly, also the growth of the patient-derived primary squamous cell carcinoma (SCC) cells was inhibited by the NF CDM matrix compared with the CAF CDM (Supplementary Fig. 3e), demonstrating that the growth-restrictive ability of NF CDM is widely applicable to different carcinomas. Similarly to the TIFF CDM, the growth restriction was specifically due to the matrix and not soluble factors as coculture of SCC or MDA-MB-231 cells with NFs or CAFs separated by a filter or conditioned medium from NFs or CAFs had no effect on proliferation (Supplementary Fig. 3f–h). In line with results obtained in breast cancer cells, JMJD1a silencing was sufficient to inhibit the proliferation of patient-derived SCC cells (Supplementary Fig. 3i). Concurrent with the ability of NF-derived CDM to downregulate JMJD1a levels, cancer cells on plastic and CAF CDM expressed high levels of JMJD1a, whereas JMJD1a was downregulated on normal CDMs (TIFF and NF; Fig. 3h), further validating the ability of normal CDM to restrict proliferation by JMJD1a downregulation.

### JMJD1a levels correlate with activated stroma within tumours

**Figure 4:**
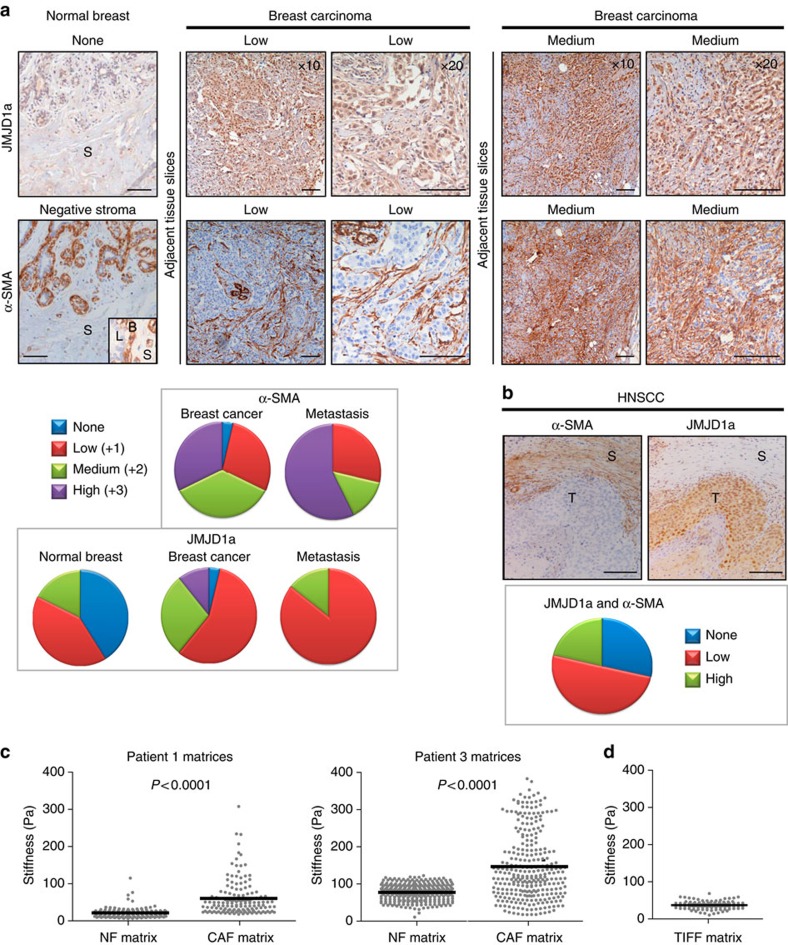
JMJD1a levels correlate with α-SMA-positive stroma in human tumours. (**a**,**b**) JMJD1a and α-SMA staining from sections of the same normal breast tissue and primary breast carcinomas (**a**) or HNSCC (**b**) tissue. For breast carcinomas, examples of the immunostaining of different expression levels and two different magnifications of the same tissue are shown. Scale bar, 200 μm. S, stroma; T, tumour. Inset in **a** highlights the α-SMA localization in basal cells in the normal mammary gland (B, basal; L, luminal). Quantification of the incidence of JMJD1a or α-SMA positivity in the analysed samples is shown. (**c**,**d**) Stiffness of the patient-derived NF or CAF (**c**) and TIFF (**d**) CDMs is expressed by the Young's modulus, which was measured using AFM indentation. Each grey dot represents individual measurement and black line indicates the mean. Non-paired *t*-test was used for statistical analysis. Non-paired *t*-test was used for statistical analysis.

The data above demonstrate that NF CDM downregulates and CAF CDM supports levels of JMJD1a. We found histologic features in human breast cancer and HNSCC tumours that are compatible with our experimental model, suggesting that the CAF matrix supports JMJD1a expression. We observed that JMJD1a expression coincides with the presence of α-SMA-positive stromal cells, which are characteristic for the tumour microenvironment^32^,^33^. Staining of 10 normal breast tissue samples, 28 primary breast cancer sections and 7 lymph node metastasis revealed that in normal breast tissues JMJD1a expression was low or absent and α-SMA was restricted to the basal mammary epithelial cells (Fig. 4a). In contrast, 27/28 of the breast cancers were JMJD1a-positive (low: 57%; intermediate: 29%; high: 11%) and 27/28 of the tumours had α-SMA-positive stroma (Fig. 4a). In addition, all metastases were positively stained for JMJD1a and α-SMA. In HNSCC patient samples, 10/14 of the cancers were JMJD1a- and α-SMA-positive (JMJD1a low: 50%; JMJD1a high: 21%). All HNSCC samples that were highly JMJD1a-positive also exhibited intense α-SMA expression in the stroma (Fig. 4b). These analyses suggest that JMJD1a is expressed in breast and HNSCC carcinomas and correlates with the presence of α-SMA-positive stroma in patients. Since increased matrix stiffness has been linked to cancer-associated stromal alterations and cell proliferation, we measured the stiffness of the patient-derived fibroblast-generated CDMs and TIFF CDM using atomic force microscopy (AFM). High indentation forces of up to 30 nN were applied. Pairwise comparison of matrices generated from fibroblasts from the same patient demonstrated that CAF-derived matrices were significantly stiffer than the normal matrix (Fig. 4c). In addition, stiffness of the TIFF CDM was similar to the NF CDMs, in line with the similar growth-inhibitory properties of the matrices (Fig. 4d). These stiffness values for NF-generated CDMs and the higher range of stiffness in the CAF CDM are highly consistent with earlier measurements on CAF-contracted collagen gels^30^ and differences in tissue stiffness observed in normal breast tissue and cancer^34^.

### JMJD1a levels and localization are regulated by stiffness

The requirement for intact ECM and the fact that normal CDM was less stiff compared with the CAF CDM suggested that matrix stiffness, in addition to matrix architecture and possibly composition, could be involved in the ability of the normal matrix to inhibit cancer cell proliferation. To test this in a controlled manner, cancer cells were grown on collagen I-coated hydrogels of varying stiffness. MDA-MB-231, HeLa and patient-derived SCC cells proliferated significantly more on stiffer supports (Supplementary Fig. 4a,b), suggesting that the lower stiffness of NF CDM is likely to contribute to its growth-restrictive properties.

**Figure 5:**
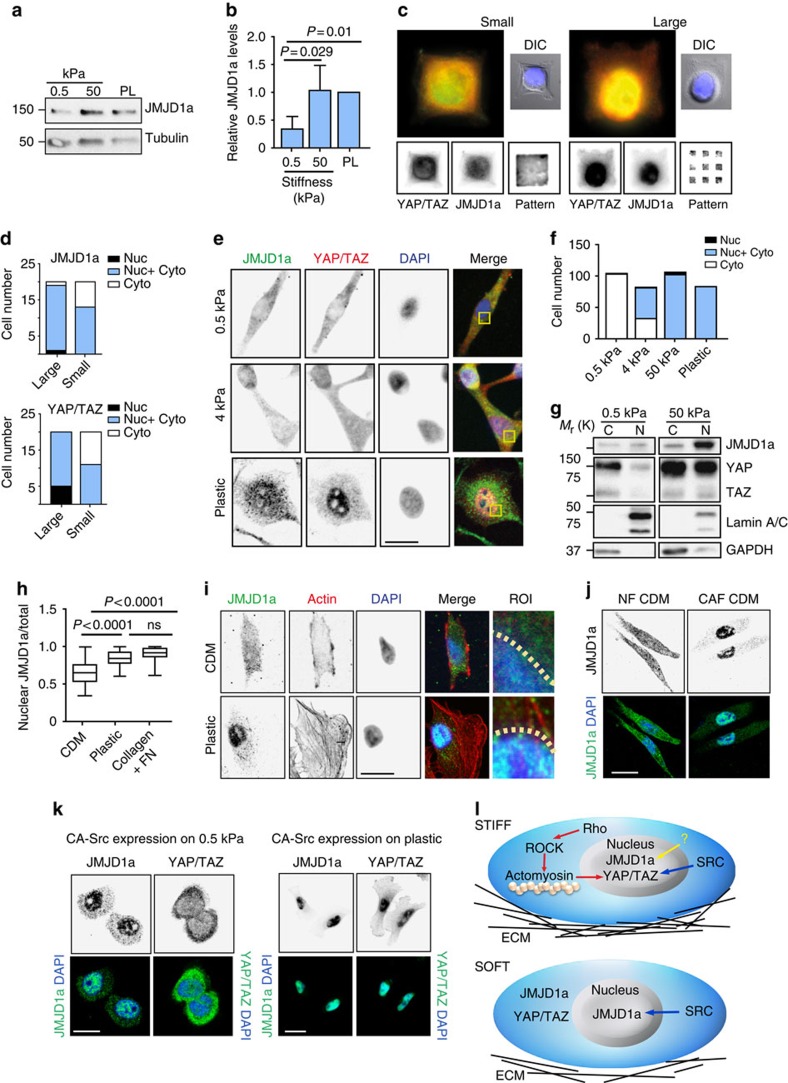
Mechanosensitive regulation of JMJD1a on soft and stiff ECM and CDM. (**a**,**b**) Representative western blot (**a**) and quantification (**b**) showing JMJD1a expression in cells plated on 0.5 and 50 kPa hydrogels and on plastic (PL). Tubulin was used as loading control, *n*=4 (mean±s.d.). (**c**) JMJD1a (red) and YAP/TAZ (green) and DAPI (blue) staining in MDA-MB-231 cells on large (800 μm^2^) and small (400 μm^2^) spreading area micropatterns (adhesive area is the same). Cell morphology is shown as DIC. (**d**) Quantification of cytoplasmic and nuclear JMJD1a and YAP/TAZ localization on small and large micropatterns. *n*(cells)=20 per pattern size. (**e**,**f**) Immunofluorescence staining showing (**e**) and quantifying (**f**) YAP/TAZ and JMJD1a localization on collagen I and fibronectin-coated hydrogels of varying stiffness (0.5, 4 and 50 kPa) and on plastic. Scale bar, 10 μm. (**g**) Representative western blot showing JMJD1a and YAP/TAZ nuclear (N) and cytoplasmic (CP) localization in cells plated on 0.5 and 50 kPa hydrogels. Lamin A/C and GAPDH were used as fractionation controls. (**h**,**i**) Immunofluorescence staining quantification (**i**) and representative images (**h**) of JMJD1a localization in cells plated on TIFF CDM, plastic or collagen and fibronectin ligands (2.5 μg ml^−1^ collagen and 2.5 μg ml^−1^ fibronectin). Nuclear localization of JMJD1a was quantified with the CellProfiler software. *n*(cells): CDM=74 cells; plastic=33 cells and collagen+FN=61. Scale bar, 10 μm. (**j**) Representative immunofluorescence images showing JMJD1a localization in MDA-MB-231 cells growing on NF and CAF CDMs for 3 days. Scale bar, 10 μm. (**k**) YAP/TAZ and JMJD1a localization in CA-SRC-expressing MDA-MB-231 cells growing on soft 0.5 kPa hydrogels or on plastic. Representative images from three independent experiments. Scale bar, 10 μm. (**l**) Model of distinct mechanotransductional regulation of YAP/TAZ and JMJD1a on soft and stiff. Red arrows indicate the pathway, which we and others have shown to regulate YAP/TAZ nuclear localization. Blue arrow indicates the SRC kinase-mediated and stiffness-dependent regulation of JMJD1a and YAP/TAZ.

JMJD1a has thus far been reported to localize to the nucleus^19^,^27^,^35^ in line with its function as a histone demethylase. However, nuclear fractionation analyses revealed that JMJD1a localizes both to the nucleus and to the cytoplasm in MDA-MB-231 cells (Supplementary Fig. 4c), suggesting that it might be shuttling between the two compartments. YAP/TAZ transcription factors are well-established mechanosensitive regulators of cell proliferation, such that stiff matrix and cell spreading support YAP/TAZ protein stability and nuclear localization^36^. To study the potential mechanosensitivity of JMJD1a, we investigated JMJD1a localization and levels under conditions known to regulate YAP/TAZ^36^. YAP/TAZ protein levels are known to be downregulated on soft supports. Interestingly, we found that also JMJD1a stability and therefore the protein levels were reduced when cells were cultured on soft hydrogels (Fig. 5a,b and Supplementary Fig. 4d).

In addition to matrix stiffness, RhoA-signalling, actomyosin contractility and cell spreading are known regulators of YAP/TAZ localization and protein stability^16^. To test whether these ques regulate JMJD1a as well, we plated MDA-MB-231 cells on micropatterns with equal total adhesive surface distributed over a variable spreading area. In cells spreading on 800-μm^2^ fibronectin-coated micropatterns (large), both JMJD1a and YAP/TAZ were predominantly nuclear compared with cells spreading on 400-μm^2^ (small) fibronectin-coated micropatterns, where both proteins were predominantly cytoplasmic (Fig. 5c,d). Accordingly, we observed that both JMJD1a and YAP/TAZ became cytosolic on lower stiffness hydrogels (0.5 kPa) as soon as the cells had fully adhered (3 h) and were increasingly nuclear on stiffer supports both in MDA-MB-231 and patient SCC cells (Fig. 5e–g and Supplementary Fig. 5a–d), whereas another nuclear protein Son was not mechanosensitive on hydrogels (Supplementary Fig. 5e). Importantly, both JMJD1a and YAP/TAZ were predominantly cytoplasmic in MDA-MB-231 cells grown on soft, normal CDM compared with their nuclear localization on plastic or collagen- and fibronectin-coated plastic (Fig. 5h,i and Supplementary Fig. 5f), and very similar regulation was observed also in the patient-derived SCC cells (Supplementary Fig. 5g). Correspondingly, JMJD1a was prominently cytoplasmic on NF CDM compared with its nuclear localization on CAF CDMs (Fig. 5j), indicating that CDM-induced regulation of JMJD1 localization and levels is similar to the previously established regulation of YAP/TAZ (Supplementary Fig. 5h).

However, unlike YAP/TAZ, JMJD1a localization was not dependent on an intact actin cytoskeleton or Rho-signalling as Blebbistatin, Cytochalasin D or ROCK inhibitor Y27632 treatment did not change JMJD1a localization, while YAP/TAZ became predominantly cytoplasmic with all these treatments (Supplementary Fig. 6a). Integrin signalling is known to be important for mechanosignalling^37^,^38^,^39^,^40^, albeit the specific requirement for integrin–ECM interaction in YAP/TAZ regulation remains controversial^41^,^42^,^43^. In order to investigate whether integrin β1 activity could regulate JMJD1a, we plated cells on integrin β1 inactivating (4B4) or activating monoclonal antibodies (12G10) and analysed JMJD1a localization in serum-free conditions. We found that locking integrins into active or inactive conformation was not sufficient to alter JMJD1a localization on soft or stiff (Supplementary Fig. 6b). Furthermore, silencing of integrin β1 from MDA-MB-231 cells had no effect on the nuclear localization of JMJD1a, suggesting that JMJD1a nuclear localization is unlikely to be dependent on a specific integrin heterodimer (Supplementary Fig. 6c). Next, we tested whether known integrin downstream effectors, Focal adhesion kinase or Rous sarcoma oncogene cellular homolog (SRC)-kinase, could regulate JMJD1a localization. We found that pharmacological inhibition of Focal adhesion kinase and SRC alone or in combination did not alter JMJD1a localization on plastic but SRC inhibition induced YAP/TAZ translocation to the cytoplasm (Supplementary Fig. 6d). However, expression of constitutively active SRC (CA-SRC) increased tyrosine phosphorylation of GFP-JMJD1a (Supplementary Fig. 6e) and was sufficient to induce nuclear JMJD1a on soft 0.5 kPa hydrogels (Fig. 5k), indicating that SRC activation is sufficient to support JMJD1a nuclear localization on soft. Interestingly, forced expression of CA-SRC did not alter YAP/TAZ localization on soft (Fig. 5k), demonstrating that stiffness-mediated regulatory pathways of YAP/TAZ and JMJD1a localization are distinct (Fig. 5i).

### JMJD1a regulates YAP/TAZ expression

**Figure 6:**
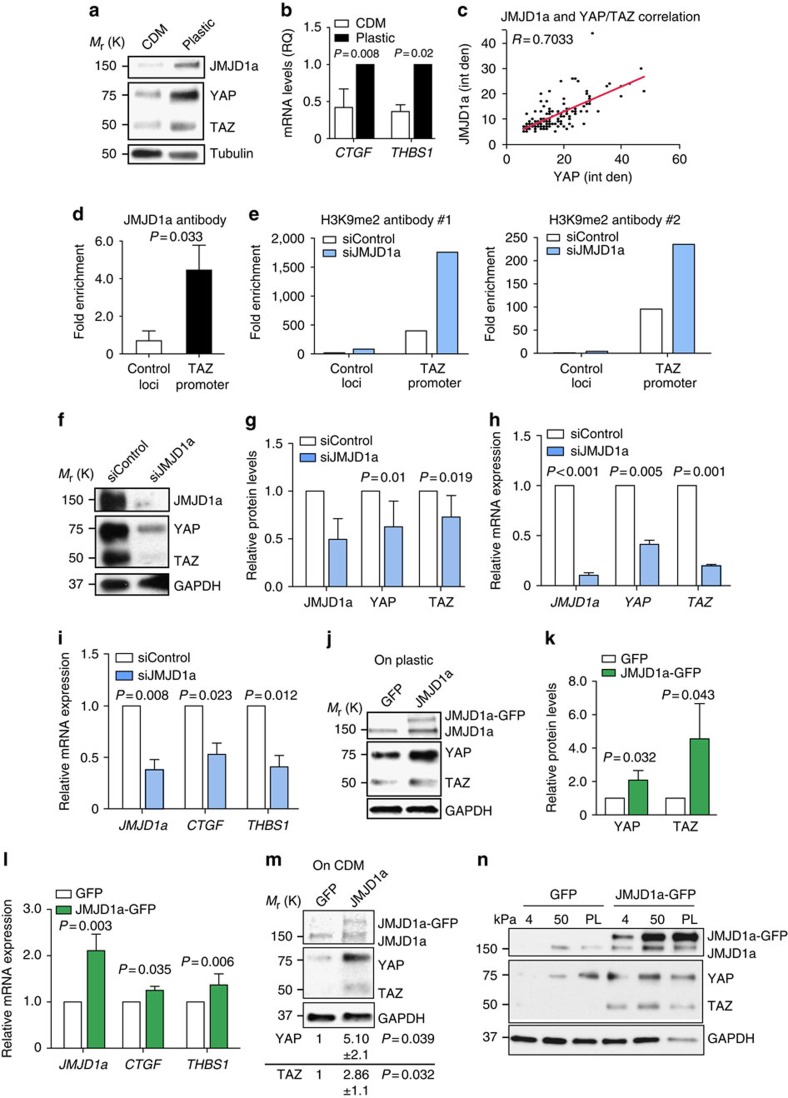
JMJD1a regulates YAP/TAZ transcription (**a**) Representative western blot showing YAP/TAZ expression in MDA-MB-231 on CDM and on plastic. (**b**) Taqman qRT–PCR of *CTGF* (*n*=4) and *THBS1* (*n*=5) mRNA levels in MDA-MB-231 cells grown on CDM or on plastic. (**c**) Quantification of JMJD1a and YAP/TAZ staining intensity from immunofluorescence images of MDA-MB-231 cells on plastic. Intensity (int den) was quantified using the CellProfiler software. *n*(cells)=146. *R*-value indicates correlation. (**d**) ChIP showing the binding of JMJD1a to the TAZ promoter. Analysis was performed by SYBR green-based detection and fold increase in signal relative to the background signal (IgG control antibody) is shown. *n*=3 (mean±s.d.). Paired *t*-test was used to calculate *P* value. (**e**) ChIP showing H3K9me2 levels on TAZ promoter of siControl and siJMJD1a_3-transfected MDA-MB-231 cells. ChIP was performed with two independent H3K9me2 antibodies. Analysis was performed by SYBR green-based detection and fold increase in signal relative to the background signal (IgG control antibody) is shown. Representative results from two independent experiments. (**f**,**g**) A representative western blot (**e**) and quantification (**f**) showing YAP/TAZ expression in JMJD1a-silenced MDA-MB-231 cells after 3 days of silencing, *n*=7. (**h**) Taqman qRT–PCR of JMJD1a (*KDM3A*), *CTGF* and *THBS1* mRNA levels in JMJD1a-silenced MDA-MB-231 cells, *n*=4. (**i**) Taqman qRT–PCR of *YAP* and *TAZ* mRNA levels in JMJD1a siRNA-transfected MDA-MB-231 cells. *n*(JMJD1a and TAZ)=4, *n*(YAP)=3. (**j**,**k**) Representative western blot (**i**) and quantification (**j**) showing YAP/TAZ expression in JMJD1a-overexpressing MDA-MB-231 cells normalized to loading control, *n*=4. (**l**) Taqman qRT–PCR of JMJD1a (*KDM3A)*, *CTGF* and *THBS1* mRNA levels in JMJD1a-overexpressing MDA-MB-231 cells, *n*=4. (**m**) A representative western blot and quantification of YAP/TAZ expression on TIFF-derived CDM in GFP control and JMJD1a-GFP-overexpressing cells after 4 days on CDM. (**n**) Representative western blot of JMJD1a and YAP/TAZ expression in GFP control and JMJD1a-overexpressing MDA-MB-231 cells plated on 4 or 50 kPa hydrogels or on PL.

JMJD1a and YAP/TAZ levels were significantly reduced on TIFF CDM (Fig. 6a) in line with their cytoplasmic translocation (Supplementary Fig. 5f), and this correlated with reduced transcription of the well-known YAP/TAZ target genes Connective tissue growth factor (*CTGF*) and Thrombospondin 1 (*THBS1*) on TIFF CDM compared with plastic (Fig. 6b). Furthermore, we observed that levels of YAP/TAZ and JMJD1a in individual MDA-MB-231 cells correlated significantly (Fig. 6c). Thus, we were interested to investigate the potential link between JMJD1a and YAP/TAZ. Chromatin immunoprecipitation (ChIP) assays revealed that JMJD1a is recruited to TAZ promoter (Fig. 6d, Supplementary Fig. 7a) and, in line with the demethylase activity of JMJD1a, transient JMJD1a silencing increased H3K9me2 methylation on the TAZ promoter (Fig. 6e and Supplementary Fig. 7a). Furthermore, JMJD1a silencing with two independent short interfering RNAs (siRNAs) reduced YAP/TAZ protein and mRNA levels in MDA-MB-231 and patient-derived SCC cells (Fig. 6f–h and Supplementary Fig. 7b–e) as well as expression of YAP/TAZ target genes in MDA-MB-231 (Fig. 6i and Supplementary Fig. 7a). Conversely to JMJD1a silencing, overexpression of JMJD1a-GFP (which localizes to the nucleus similarly to endogenous JMJD1a on plastic, Supplementary Fig. 7f) increased YAP/TAZ levels (Fig. 6j,k) as well as *THBS1* and *CTGF* gene expression (Fig. 6l). Importantly, overexpression of JMJD1a was sufficient to increase YAP/TAZ levels even on TIFF-derived CDMs (Fig. 6m) and 4 kPa hydrogels (Fig. 6n), where YAP/TAZ protein-level stability is compromised because of increased cytoplasmic localization and degradation^36^. However, overexpression of wild-type or active YAP mutant (YAP-5SA (ref. 44)) alone was not sufficient to rescue proliferation on MDA-MB-231 cells grown on TIFF-derived CDMs or in JMJD1a-silenced cells, suggesting that additional JMJD1a target genes contribute to the CDM-induced growth inhibition (Supplementary Fig. 7g,h).We also found that silencing of JMJD1a had no effect on either YAP/TAZ nuclear localization (Supplementary Fig. 8a) or phosphorylation of LATS1/2 (Supplementary Fig. 8b), which is a negative regulator of YAP/TAZ protein stability. Furthermore, we could not detect increased YAP (S-127) phosphorylation, which is associated with reduced stability of YAP, upon JMJD1a silencing (Supplementary Fig. 8c) and protein stability of YAP/TAZ was not reduced upon JMJD1a silencing, suggesting that JMJD1a regulates YAP/TAZ on the transcriptional level (Supplementary Fig. 8d). This further demonstrates that JMJD1a is a previously undescribed transcriptional activator of YAP/TAZ expression, and that forced expression of JMJD1a can support YAP/TAZ levels even on soft substrates because of its ability to increase the transcription of YAP/TAZ.

### JMJD1a and YAP/TAZ expression correlates in human cancer

We found that JMJD1a and YAP/TAZ expression correlated also in human carcinomas. In a large cohort of primary breast tumours^45^, JMJD1a and YAP/TAZ (the YAP antibody recognizes both transcription factors) were strongly associated with several commonly assessed clinicopathological prognostic factors (Supplementary Tables 1–4 and Supplementary Note 1). In all, 689 (94.3%) and 262 (35.8%) out of the 731 tumours available for JMJD1a staining had positive cytoplasmic and nuclear staining, respectively, and 645 (86.5%) and 514 (68.9%) out of the 746 cancers available for YAP/TAZ staining had positive cytoplasmic and nuclear YAP/TAZ expression.

**Figure 7:**
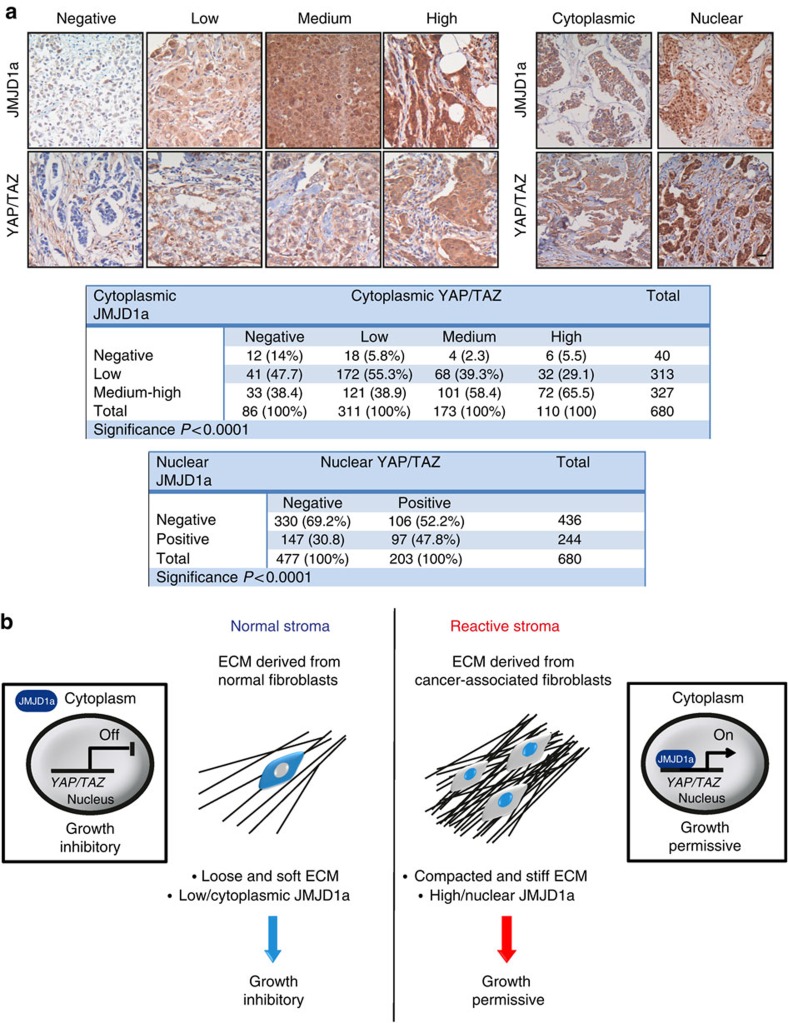
JMJD1a and YAP/TAZ expression correlates in human carcinomas. (**a**) Example images for the scoring of immunohistochemical stainings for JMJD1a and YAP/TAZ expression from primary breast cancer tissue samples. Scale bar, 100 μm. The tables show the association between cytoplasmic and nuclear expression of JMJD1a and YAP/TAZ. The *χ*^2^-test was used for the statistical analyses. (**b**) A proposed model for the normal and cancer-associated matrix-induced control of cancer cell proliferation via mechanosensitive regulation of JMJD1a and transcriptional regulation of YAP/TAZ.

Fully in line with their correlated expressions *in vitro*, we found that JMJD1a and YAP/TAZ levels significantly correlated in clinical tumour samples available for both stainings (Fig. 7a). Furthermore, nuclear localization of YAP/TAZ was significantly associated with nuclear JMJD1a in tumours (Fig. 7a). A similar correlation was found also on the mRNA level. Integrated data from multiple gene-expression-profiling studies of breast ductal carcinoma (720 patients) and oral and skin SCC (47 patients) showed a significant positive correlation between JMJD1a (*KDM3A*) and TAZ (*WWTR1*) gene expression (Supplementary Fig. 9). Taken together, these data reveal a previously unknown mechanosensitive relationship between JMJD1a and YAP/TAZ expression both *in vitro* in cancer cell lines and in a large number of clinical patient samples from the same cancer types.

## Discussion

We describe a new mechanism of ECM-mediated control of cancer cell proliferation. Our results indicate that epigenetic modifier JMJD1a is a mechanosensitive regulator of the transcription of many genes, including *YAP* and TAZ *(WWTR1)* and of cancer cell proliferation (Fig. 7b). In cancer, changes in ECM composition and mechanical properties of the tumour microenvironment are the focus of intense research, as matrix stiffening and appearance of CAFs in the tumour stroma correlate with cancer progression and metastasis^22^,^46^. However, the mechanisms of how normal tissue could function to suppress or restrict tumour growth are much less well understood. We propose that ECM generated by NFs is growth-restrictive to cancer cells *in vitro* and has the capacity to inhibit the growth of malignant carcinomas *in vivo*. Mechanistically, the soft normal ECM triggers the translocation of histone demethylase JMJD1a from the nucleus to the cytoplasm. This is followed by JMJD1a downregulation on the protein and mRNA levels as well as a downregulation of proliferation-inducing genes, including *YAP*/TAZ *(WWTR1)*. Thus, our results define an unprecedented level of regulation of cancer cell proliferation, where the mechanosensitive control of JMJD1a regulates its nuclear availability to regulate transcription of proliferative genes.

By comparing CDMs generated by NFs and CAFs from the same individual, we found that normal CDM, but not CAF CDM, had a strong growth-inhibitory influence on primary patient-derived HNSCC cells. CAF-derived CDM exhibited altered architecture as well as increased stiffness compared with the NF-derived CDM, in line with what has been demonstrated for carcinomas in patients^34^,^47^. Thus, it appears that normal stroma possesses tumour-suppressive functions that are lost in the reactive cancer-associated stroma. We found that tumour-suppressive capacity of the normal stroma is connected to stiffness but is likely to be mediated by changes also in matrix architecture and composition. This observation could be linked to the important but largely unexplored fact that, even though cancer incidences are increasing globally, most people never get cancer. Pathologists frequently observe numerous microscopically detectable cancerous foci in the mammary gland or prostate of people who never develop advanced clinical cancer^48^. Furthermore, several studies (reviewed in ref. 48) have demonstrated that normal tissue has the capacity to restrict the growth of cancer cells and to determine the cancer incidence of transplanted normal tissue.

JMJD1a is regulated by hypoxia^28^,^49^; however, its regulation under normoxic conditions has not been investigated. We find that matrix stiffness regulates the subcellular localization of JMJD1a and that on soft matrices JMJD1a is cytoplasmic and becomes downregulated. JMJD1a has been found to be upregulated in several cancer types^50^,^51^ and to be important for cancer cell proliferation^52^. In human bladder carcinomas, JMJD1a overexpression could already be detected at early-stage carcinomas before the generation of hypoxic conditions^27^. These data suggest that JMJD1a contributes to cancer progression even under normoxia and that JMJD1a levels are regulated early in cancer progression. We find in clinical samples that a vast majority of human breast carcinomas express elevated levels of JMJD1a compared with normal tissue. Furthermore, the stroma in these tumours is positive for the CAF marker α-SMA. Thus, JMJD1a expression also correlates with stromal changes *in vivo*.

Breast cancer expression of JMJD1a and YAP/TAZ were strongly associated with several unfavourable prognostic factors in the breast cancer cohort^45^, suggesting clinical relevance. Tumour JMJD1a and YAP/TAZ expression did not, however, influence survival, and their expression was not associated with an increased frequency of axillary nodal metastases. Therefore, while JMJD1a and YAP/TAZ favour cancer proliferation, their expression might not contribute to metastasis.

Regulation of the YAP/TAZ pathway has been intensely investigated in development and cancer^53^. However, the focus has been predominantly on how YAP/TAZ nuclear localization and protein stability are controlled and how YAP and TAZ regulate the transcription of their target genes together with DNA-binding proteins of the TEAD (TEA/ATTS domain) family^54^. We find that JMJD1a regulates YAP/TAZ transcription in a stiffness-sensitive manner, and that forced expression of JMJD1a is sufficient to support YAP/TAZ levels also on soft substrates. This is to the best of our knowledge the first mechanistic demonstration for mechanosensitive regulation of an epigenetic regulator enzyme as well as of YAP/TAZ on the transcriptional level.

## Methods

### Cell lines and cell culture

SCC cell lines and CAFs were isolated from different parts of head and neck region (Supplementary Fig. 1a). The UT-SCC-54A (Patient #2) cell line was established from a primary T2N0M0 Grade 1 tumour of the buccal mucosa Grade 1. The donor (non-smoker) did not excessively consume alcohol. The UT-SCC cell line was established using the explant method from primary, recurrent or metastatic tumours. The fibroblasts were removed by selective trypsinization as previously described in ref. 55. The CAF population was identified by SMA-α expression by western blotting. NFs were isolated from a normal, non-cancerous region. MDA-MB-231 human breast adenocarcinoma cells (American Type Culture Collection, ATCC), primary fibroblasts and SCC (UT-SCC-54A) cells were maintained in DMEM (4500, mg l^−1^ glucose, Sigma) containing 1% non-essential amino acids (Sigma), 1% L-Glutamine (Gibco) and 10% fetal bovine serum (FBS). HeLa cells (ATCC) were maintained in DMEM (1000, mg l^−1^ glucose) supplemented with 10% FBS and 1% L-glutamine. The usage of human tissue to derive cell lines was approved by the Finnish national authority for medicolegal affairs (Dnro 889/04/047/08) and regional ethics committee of University of Turku (Dnro 146/2007).

### Cell-derived matrices

CDMs were prepared as described in ref. 20. Coverslips were coated with 0.2% gelatin for 60 min at 37 °C (Sigma G1393 in PBS), followed by crosslinking with 1% glutaraldehyde for 30 min at room temperature (RT). Crosslinker was quenched with 1 M glycine for 20 min at RT. Gelatin-coated coverslips were incubated with medium before seeding 50,000 cells on coverslips. Ascorbic acid treatment (50 μg ml^−1^) was started when the cell layer was confluent. Ascorbic acid-containing medium was changed every day for 10 (TIFFs)—or 21 days (primary fibroblast cell lines). After ascorbic acid induction, cells are removed by extraction buffer treatment (20 mM NH_4_OH, 0.5% Triton-X in PBS). Remaining DNA was removed by 10 μM DNAse treatment for 1 h at 37 °C.

### Proliferation assays

Proliferation assays for the MDA-MB-231 and HeLa cells were performed using Incucyte-FLR or Incucyte-ZOOM live-cell microscopy incubator (Essen Bioscience). In all, 5,000 of the cancer cells to be analysed (GFP-positive HeLa or MDA-MB-231 cells) were plated on 24 wells (on CDM or on plastic) and imaged hourly over several days. Stably GFP-positive MDA-MB-231 and HeLa cells were used throughout to facilitate determining the cell number at the indicated time points on CDM. The doubling times were calculated using GraphPad (nonlinear regression curve−exponential growth equation; *Y*=*Y*0*exp(k*X)]. Since the SCC cells were not GFP-positive, their proliferation was analysed by counting the nuclei (4,6-diamidino-2-phenylindole (DAPI) staining). Cells were plated on CDMs or on plastic in identical numbers. After 4 days, the cells were fixed and stained with DAPI. Images were taken with a × 4 objective. Four images were taken from each well and each condition had three to four wells. The amount of nuclei/image was quantified with the CellProfiler software.

### Illumina microarray

The Illumina gene expression data were normalized using the quantile normalization method in Bioconductor (http://www.bioconductor.org) and log-transformed (base 2). Log ratios of the intensities were calculated between the paired samples: ECM versus plastic (*n*=2 pairs) and ECM to plastic versus plastic (*n*=3 pairs) in both MDA-MB-231 and HeLa samples. Systematic differences in gene expression between the CDM and CDM to plastic conditions were identified using the rank product algorithm (Bioconductor RankProd package) separately for the MDA-MB-231 and HeLa cell lines. Genes with false discovery rate below 0.05 and change in the same direction in both cell lines were considered as differentially expressed.

### Quantitative RT–PCR

For quantitative reverse transcriptase (qRT–PCR), total RNA was extracted using an RNAeasy Mini Kit (Qiagen) and converted to cDNA using a high-capacity cDNA Reverse Transcription Kit (Applied Biosystems) according to the manufacturer's instructions. TaqMan probe-based quantitative real-time PCR was analysed using Real-Time PCR HT7900 (Applied Biosystems). Glyceraldehyde-3-phosphate dehydrogenase (*GAPDH*) was used as endogenous control. The qPCR primers and universal probe library probes used are listed in Supplementary Table 5.

### mRNA sequencing

For the mRNA sequencing, NFs and CAFs were collected from 10-cm cell culture dishes, lysed in RLT lysis buffer (Qiagen) and RNA was isolated using the NucleoSpin Kit (Macherey-Nagel). Sequencing was performed with Illumina HiSeq2500 instrument using single-end sequencing chemistry with 50-bp read length.

### Chromatin immunoprecipitation

In order to collect the cells, the growth medium was removed and the culture was washed three times with PBS. We collect minimum two million cells per antibody. Protein–chromatin complexes were crosslinked with 1% paraformaldehyde (PFAH) for 10 min at RT (in case of H3K9me2). For JMJD1a, ChIP cells were crosslinked with disuccinimidyl glutarate (Sigma) for 45 min followed by 10 min incubation in 1% PFAH. Disuccinimidyl glutarate was used for JMJD1a ChIP since crosslinkers with longer linker arms have been used for JMJD1a ChIP^49^. Crosslinking reaction was stopped with 0.125 M Glycine by 5-min incubation. Cells were detached with scraper, collected by centrifugation (300*g*, 10 min) and washed once with pre-lysis buffer I (10 mM EDTA, 0.5 mM EGTA, 10 mM HEPES and 0.25% Triton X) and once with pre-lysis buffer II (1 mM EDTA, 0.5 mM EGTA, 10 mM HEPES and 100 mM NaCl). Cells were lysed by suspending the pellet in 1 ml of lysis buffer (10 mM EDTA, 50 mM Tris-HCl and 0.5% SDS, protease inhibitors) and incubating samples for 10 min on ice. DNA was fragmented by 4 × 5 min sonication (30 s on, 30 s off). Sheared chromatin was spun down at 10,000*g* for 1 min at 4 °C. Chromatin was aliquoted so that ∼100 μg of chromatin was used per antibody. Dilution buffer (1% Triton-X100, 2 mM EDTA, 150 mM NaCl and 20 mM Tris-HCl) was used to adjust the total volume to 600 μl s^−1^. Chromatin was pre-cleared by adding 50 μl of Dynabeads (10003D, Life Technologies) and incubating for 1 h at 4 °C on rotation. Beads were removed and 5 μg of JMJD1a (sc-376608, Santa Cruz), H3K9me2 antibody #1 (ab1220, Abcam) H3K9me2 antibody #2 (C15410060, Diagenode) or mouse control IgG (sc-2025, Santa Cruz) antibody was added per sample, followed by overnight incubation at +4 °C on rotation. In order to collect the immunocomplexes, 50 μl of Dynabeads was added per sample followed 2 h incubation at +4 °C on rotation. Washes were performed once with low-salt wash buffer (2 mM HEPES, 150 mM NaCl, 1% Triton X, 1 mM EDTA and 20 mM Tris-HCl), four times with high-salt wash buffer (50 mM HEPES, 500 mM NaCl, 0.1% SDS, 1% Triton X and 1 mM EDTA) and twice with TE buffer (10 mM Tris-HCl and 1 mM EDTA pH 8.0). DNA was collected to elution buffer by 15-min incubation at RT, and proteins were removed by Proteinase K incubation at 55 °C overnight. DNA was isolated by standard phenol–chloroform–isoamyl extraction (25:24:1 phenol–chloroform–isoamyl pH 7.8). Real-time PCR was carried out using 5 μg of DNA per reaction and SYBR green-based detection (Qiagen RT2 SYBR Green ROX qPRC Mastermix cat: 330520) according to the manufacturer's instructions. EpiTect ChIP qPCR Primer Assay For Human WWTR1 (TAZ) was used: NM_015472.3 (+)01Kb: GPH1023327(+)01A for JMJD1a ChIP and NM_015472.3 (−)04Kb: GPH1023327(−)04A for H3K9me2 ChIP. Negative control primers #1 and #2, which recognize untranslated genomic regions, were used (Control#1 forward 5′-CTGTACCTGGGGTTCATTCAT-3′ and reverse 5′-CAGTAAGCCGTTCACTCTCACA-3′; Control#2 forward 5′-ATCACACTGCAAAAATCCAGAA-3′ and reverse 5′-TCACTTCTTTAACTGGCCTTGA-3′). Fold enrichment was calculated by subtracting the background signal (Ct (IP)−CT(IgG control)) and calculating the fold enrichment (2^−DDCt^).

### Western blotting and phosphotyrosine pull-downs

Standard western blotting techniques and Amersham ECL Plus Western blotting reagent were used. Following antibodies were used: JMJD1a (12835-1-AP, Proteintech, USA, 1:1,000), YAP/TAZ (sc-101199, Santa Cruz Biotechnology, USA, 1:500), GAPDH (5G4, HyB test 1:5,000), Tubulin (12G10, Hybridoma bank, 1:5,000), Lamin A/C (sc-7292, Santa Cruz Biotechnology, 1:1,000), H3K9me2 (#7658, Cell Signaling, 1:1,000), Histone 3 (#4499, Cell Signaling, 1:1,000), actin (clone AC-74, Sigma, 1:1,000), alpha-SMA (A2547, Sigma, 1:1,000) and antiphosphotyrosine antibody (APY03, Cytoskeleton, 1:1,000). For the phosphotyrosine pull-downs, MDA-MB-231 cells co-transfected with GFP-JMJD1a and CA-Src or empty vector were lysed and subjected to pulldown with beads of APY03 covalently coupled to sepharose (Anti-Phosphotyrosine Affinity Beads # APY03-Beads (Cytoskeleton) according to the manufacturer's instructions. The pulldowns and cell lysate were resolved on SDS–PAGE and subjected to western blot analysis with anti-GFP antibody (Abcam #1218).

Uncropped scans of the most important blots are provided as Supplementary Fig. 10.

### Immunofluorescence

Cells were fixed with 4% PFAH for 10 min at RT and simultaneously permeabilized and blocked with 0.3% Triton in 30% horse serum (Gibco) for 10 min at RT. Following antibodies and antibody dilutions were used: JMJD1a (12835-1-AP, Proteintech, 1:100), YAP/TAZ (sc-101199, Santa Cruz Biotechnology, 1:75), JMJD1a (sc-376608, Santa Cruz Biotechnology, 1:100), Atto-Phalloidin-647N (65906, Sigma, 1:200), Collagen I (NB600-408, Novus, 1:100) and Fibronectin (F3648, Sigma, 1:400).

### Microscopy and image analysis

Immunofluorescence stainings were imaged with Zeiss spinning disc confocal (Orca-ER camera (Hamamatsu Photonics), Plan-Neofluar × 40or × 63 oil/1.4 numerical aperture (NA) objective (Carl Zeiss)) or with Zeiss LSM780 laser scanning confocal (× 63 water/1.4 NA objective (Carl Zeiss)). Nuclear localization of JMJD1a was quantified with the CellProfiler image analysis software. The nucleus was defined with the DAPI staining. For JMJD1a nuclear localization, total JMJD1a and nuclear JMJD1a were quantified.

### Linescan analysis of CDMs

Fibre organization of Collagen 1 and fibronectin-stained CDMs were analysed in ImageJ by drawing a line along the image (three lines per image, 10 images in total). Staining intensity was quantified along the line (→*analyse→blot profile*). *x* and *y* coordinates were exported (*→list*) and exported to GraphPad. Graph displays the average and s.d. of staining intensity along the line.

### Atomic force microscopy

NF- and CAF-derived matrices were grown as described above. The stiffness of CDMs was evaluated with JPK AFM with CellHesion module (JPK Instruments) on Zeiss LSM510 microscope (Carl Zeiss Microscopy). Silicon AFM probes with 1 N m^−1^ cantilever and spherical 45-μm polystyrene particle tip (Novascan Technologies) were used on force measurements, where Young's module was detected. During the measurements, matrices were kept in PBS at 37 °C using the BioCell module (JPK). Before the force measurements, AFM cantilevers were calibrated with the instrument's calibration programme by measuring deflection sensitivity and spring constant. Force measurements of matrix samples were performed on three conditions of a matrix using four random locations per matrix and grids of 5x5 on each location. As control for approach measurements, the region of interest was visualized with a charge-coupled device camera, mounted on the microscope. Probe extension was carried out with the following settings: travelling distance of 50 μm, speed 5 μm s^−1^ and sampling rate 205 Hz. We obtained force distance curves by indenting with forces up to 30 nN (maximal indentation <20 μm in all experiments). The gel thickness was with ∼10–15 μm sufficiently thick to assume a Hertz model^56^, and the fits used a Nelder–Mead algorithm to determine the Young's modulus and the touching point. The Hertz model did fit the full data range with a high confidence (*R*^2^>0.95 for 88% of the measurements).

### Scanning electron microscopy

Matrices were fixed with 2% glutaraldehyde in 0.1 M NaCac buffer, pH 7.4 for 20 min and washed twice with NaCac buffer. Samples were sonicated for 30–60 min at RT in 1% OsO_4_ in 0.1 M NaCac and washed twice with 0.1 M NaCac buffer. Matrices were dehydrated by 3 min incubation once with 50%, once with 70% and once with 96% EtOH (A) and finally twice with absolute EtOH (Aa) for 5 min. Finally, matrices were covered with hexamethyldisilazane. Coverslips were mounted to aluminium specimen stubs using double-sided graphite tape and addition of one drop of graphite glue to the edge of the coverslip.

### Hydrogels

Hydrogels of various stiffnesses were ordered from Marigen Life Technologies. Gels were coated for 1 h at 37 °C with fibronectin-collagen I mix (2.5 μg ml^−1^ each) before use.

### Nuclear fractionation

In order to separate the nucleus from the cytoplasmic fraction, cells were scraped in 1 ml PBS (from 6 cm dishes) and collected by centrifugation. The cell pellet was suspended in 200 μl of cold PBS containing 0.1% NP-40 followed by 30 s of centrifugation at 10,000*g* and 4 °C. The supernatant was collected and marked as cytoplasm (C). The pellet was resuspended into 1 ml of cold PBS-0.1% NP-40 buffer, and centrifugation was conducted as described earlier. The supernatant was discarded and the nuclear pellet was suspended in 20 μl of lysis buffer. The same protein amount was then loaded on 4–20% SDS–PAGE gel. Lamin A/C was used as a nuclear and GAPDH as a cytoplasmic fraction marker.

### Transfections

siRNA transfections were performed using Lipofectamine RNAiMAX transfection reagent according to the manufacturer's instructions. Allstars negative control siRNA from Qiagen (siControl_1) and non-targeting siRNA from Dharmacon (siControl_2) were used as controls. Three independent siRNAs were used to silence JMJD1a: JMJD1a_1 custom siRNA (sense: 5′-GUCUAUGUGGGAAUUCCCA-3′, antisense: 5′-UGGGAAUUCCCACAUAGAC-3′) was ordered based on previous publication^27^ from Sigma. Dharmacon JMJD1a siRNA (JMJD1a_2) was used to validate the results. Third siRNA (siJMJD1a_3; sense 5′-GCAAUUGGCUUGUGGUUACUU-3′ and antisense 5′-GUAACCACAAGCCAAUUGCUU-3′) was ordered based on previous publication^57^ from Sigma. eGFP-JMJD1a (EX-T3698-M29) constructs were ordered from GeneCopoeia. pQCXIH-Myc-YAP (Addgene plasmid #33091) and pQCXIH-Myc-YAP-5SA (ref. 44; Addgene plasmid #33093) were gifts from Kunliang Guan and constitutively active Src pLNCX chick src Y527F (Addgene plasmid # 13660) was a gift from Joan Brugge.

### Proliferation assays using YAP overexpression

Overall, 100,000 MDA-MB-231 cells were plated on CDM or plastic overnight. Next day, cells were transfected with control, YAP or YAP-5SA plasmid; the plate was placed in Incucyte-ZOOM live-cell incubator and proliferation was monitored over several days. Alternatively, MDA-MB-231 cells were transfected with control or JMJD1a siRNA for 48 h. siControl and siJMJD1a cells were trypsinized, and equal amount of cells were plated on 24-well plates. Next day, the cells were transfected with control, wt-YAP or YAP-5SA, and proliferation was monitored in Incucyte-ZOOM over several days.

### Matrix solubilization

Matrices were prepared as described above. In order to solubilize matrix proteins, the matrices were incubated in buffer containing 5 M guanidine, 10 mM dithiothreitol (DTT) and 5 mM phenylmethanesulfonylfluoride (PMSF) and incubated for 10 min at RT. Dissolved matrices were replated on 24-well plates by 1 h incubation at 37 °C. The wells were washed extensively with PBS.

### Coculture experiments

Overall, 5,000 SCC cells were plated on 24-well plates. Next day, 10,000 fibroblasts were plated inside the Transwell inserts (3 μm pore polycarbonate membrane, Corning). In addition, the amount of SCC cells (day 0 time point) was measured with WST-1 staining before adding the fibroblasts. The WST-1 reagent (Roche) was diluted 1:10 in full medium, added to the cells and incubated at 37 °C for 30 min. Absorbance at 450 nm was measured using the Multiscan Ascent plate reader (Thermo Scientific).

### Tumour growth assay in eggs

Fertilized chicken eggs were incubated as previously described in ref. 57. Shortly, the eggs were washed and the development was started by placing the eggs in 37 °C incubator. On day 3 of development, a small hole was made in the eggshell to drop the CAM. On developmental day 10, a plastic ring was placed on the CAM and one million either control or JMJD1a siRNA-transfected MDA-MB-231 cells were implanted inside the ring in 20 μl of 50% Matrigel. After 3 days, tumours were imaged and dissected. Tumours were fixed with 4% PFA overnight and processed for paraffin sections.

### Orthotopic breast tumour growth assay

MDA-MB-231 cells were transfected with control and JMJD1a siRNA for 3 days (first transfections) and 24 h (second transfection) before the injection. Two million siControl or siJMJD1a were injected into the abdominal fat pads of 6–week-old virgin female nude mice (NOD.SCID (from Envigo)). Cells were injected into both sides so that siControl cells were on one side and siJMJD1a on the other side. Altogether, 19 mice were used. Ten mice had control tumours on left (and siJMJD1a tumours on right) and nine had control cells on the right (and siJMJD1a tumours on the left). Tumour growth was measured and tumours were collected 8 days after the injection. All animal experiments were ethically assessed and authorized by the National Animal Experiment Board (ESAVI/7522/04.10.03/2012) and in accordance with The Finnish Act on Animal Experimentation.

### Clinical samples and immunohistochemistry

Normal breast tissue and cancerous primary tumour tissues with their lymph node metastasis counterparts were collected from archives of the Department of Pathology, Helsinki University Central Hospital. Haematoxylin–eosin-stained tissue sections were reviewed, and tissues representative for primary tumour and corresponding nodal metastases or healthy breast tissues were selected for the study. A clinical breast cancer series from the FinHer study was collected from patients with axillary node-positive or high-risk node-negative tumours and from those who had undergone a breast surgery with auxiliary node dissection or sentinel node biopsy for invasive breast carcinoma as described in details elsewhere^45^. Tissue microarrays were constructed from tumour-representative tissue regions. Patients, who participated in the FinHer study, provided written informed consent for research use of tumour tissue. An ethics committee at the Helsinki University Central Hospital has approved the study (HUS 106/13/03/02/2015). Cancerous primary tumour tissues from patients with HNSCC were collected from archives of the Department of Pathology, Turku University Central Hospital.

Standard immunohistochemistry techniques were applied to detect JMJD1a, YAP and α-SMA expression in tissue sections. Briefly, endogenous peroxidase activity was blocked in 1% hydrogen peroxidase in deparaffinized tissues, and heat-induced epitope retrieval was performed in sodium citrate by using 2100 Antigen Retriever (Aptum Biologics Ltd., UK). Primary antibodies were diluted in Normal antibody Diluent (Immunologic, the Netherlands) and incubated at 4 °C overnight for JMJD1a and YAP (12835-1-AP, Proteintech, dilution 1:100; 63.7, Santa Cruz Biotechnology, dilution 1:250, respectively) or 1 h at RT for α-SMA (clone 1A4, Dako, Denmark, dilution 1:200). Binding of the primary antibody was detected and visualized by using N-Histofine Simple Stain MAX PO Kits (Nichirei Biosciences Inc., Tokyo, Japan) and 3,3′-diaminobenzidine (ImmPACT DAB, Vector Laboratories, Burlingame, CA, USA) following the manufacturers' recommendations. Cytoplasmic JMJD1a and YAP expression was classified into four groups (negative, low, intermediate and high) depending on how strong the staining intensity was detected on the majority of tumour cells, whereas nuclear staining was considered as positive whenever more than 10% of tumour cell nuclei showed protein expression.

Frequency tables were analysed using the *χ*^2^-test. Distant disease survival was calculated from the date of randomization to the date of breast cancer recurrence outside of the locoregional region or to the date of death, whenever death preceded distant recurrence. Overall survival was calculated from the date of randomization to the date of death. Survival was analysed using the Kaplan–Meier method, and survival between groups was compared with the log-rank test. All *P* values are two-sided.

### Statistical analyses

The GraphPad programme was used for all statistical analyses. Student's *t*-test (paired, two-tailed) was used in most cases. Non-parametric Mann–Whitney test was used when two unpaired groups were compared and normality could not be tested (because of a too small data set (*n*<8)). Unpaired *t*-test was used when normality could be tested. Normality was tested by D'Agostino and Pearson omnibus normality test.

### Adhesion assay

We have used an xCELLigence Real-Time Cell Analysis instrument in order to measure cell adhesion on solubilized matrices. Briefly, CDMs derived from NFs or from CAFs were solubilized with Guanidine buffer (5 M guanidine, 10 mM DTT and 5 mM PMSF) for 15 min at 37 °C. In all, 50 μg ml^−1^ of each solubilized CDM or 0.2% BSA was plated on of 96-well E-plates, incubated for 1 h at 37 °C, washed with PBS and blocked with 0.2% BSA in PBS for 30 min at 37 °C. Overall, 100,000 MDA-MB-231 cells were added to each well right before the measurement was started. The cell index was measured every 3 min for 2 h and every 10 min for another 2 h. Student's *t*-test (paired, two-tailed) was used to test for statistical significance.

### Data availability

The mRNAseq and Illumina data have been deposited in the Gene Expression Omnibus (GEO) under the accession codes GSE83314 and GSE83366, respectively. All relevant data are either contained in the paper or supplementary files or are available from the authors.

## 

## Additional information

**How to cite this article:** Kaukonen, R. *et al*. Normal stroma suppresses cancer cell proliferation via mechanosensitive regulation of JMJD1a-mediated transcription. *Nat. Commun.* 7:12237 doi: 10.1038/ncomms12237 (2016).

## Supplementary Material

Supplementary Information Supplementary Figures 1-10, Supplementary Tables 1-4 and Supplementary Note 1

Supplementary Data 1 Gene expression dataset
