# Supplementary material for: Normal stroma suppresses cancer cell proliferation via mechanosensitive regulation of JMJD1a-mediated transcription
Source: Nat Commun. 2016 Aug 4;7:12237. doi: 10.1038/ncomms12237 (PMC4976218; doi:10.1038/ncomms12237)
Supplement: Supplementary Information — Supplementary Figures 1-10, Supplementary Tables 1-4 and Supplementary Note 1 [file ncomms12237-s1.pdf]

Supplementary figures, figure legends, supplementary note and supplementary tables.

Supplementary Figure 1

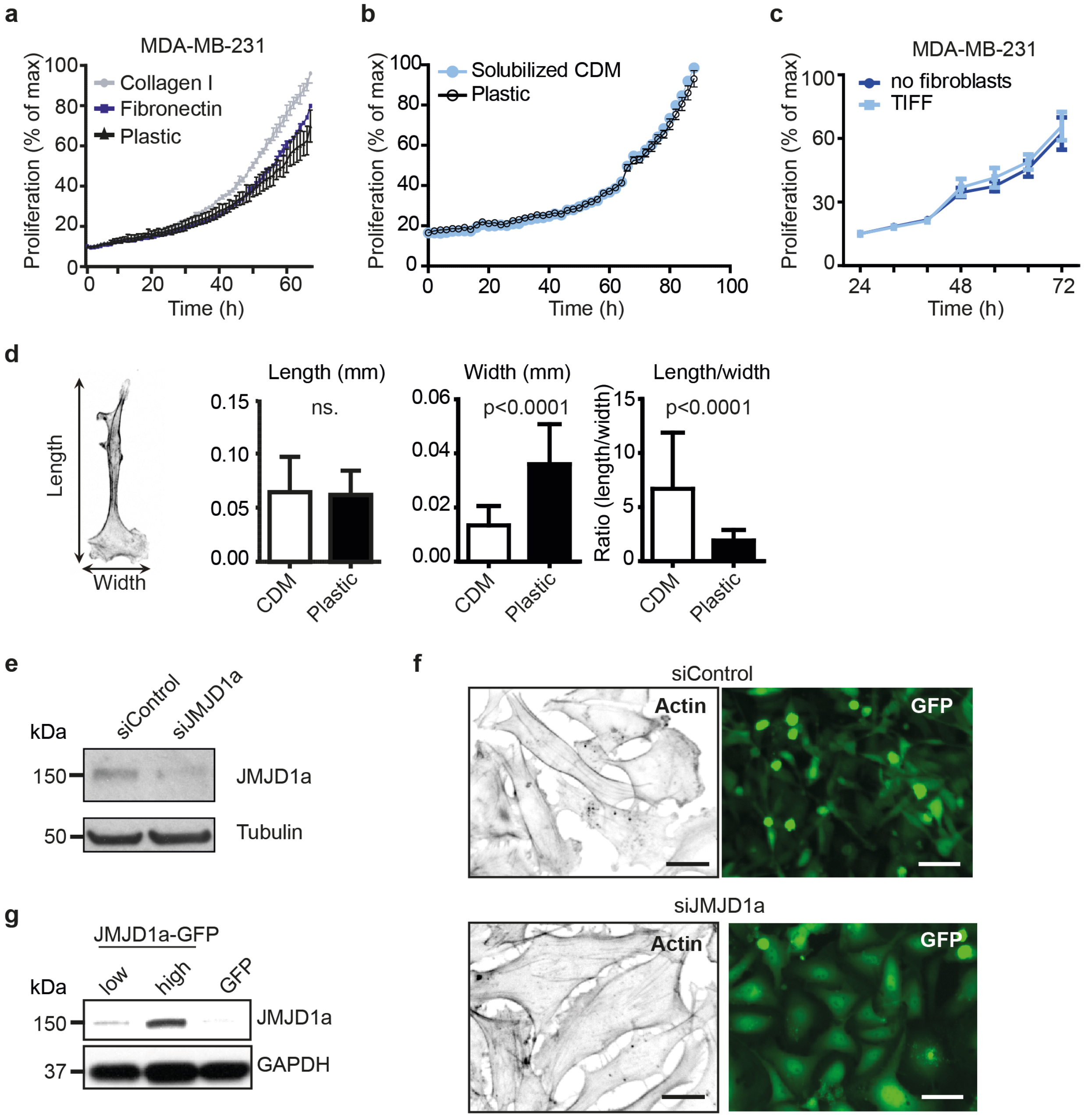

Supplementary Figure 1. Intact CDM and JMJD1a regulate cancer cell proliferation.

(a) Proliferation of MDA-MB-231 cells on collagen I or fibronectin matrix ligands compared to cells grown on plastic. **(b)** Proliferation of MDA-MB-231 cells growing on wells coated with solubilised TIFF CDM. **(c)** Proliferation of MDA-MB-231 cells in either conditioned TIFF medium or normal full-medium (no fibroblasts). (a-c) Proliferation was examined with Incucyte^{TM} -FLR by analysing the number of cells. The quantification of relative cell number over time is shown (mean±s.e.m.). **(d)** Quantification of width and length of MDA-MB-231 cells which were cultured on TIFF-CDM or on plastic. n(CDM)=31 cells, n(plastic)=30 cells. **(e)** Western blot showing JMJD1a expression in control and JMJD1a siRNA-transfected MDA-MB-231 cells. (**f**) Morphology of control or JMJD1a-silenced MDA-MB-231 cells (3 days) growing on plastic. Actin staining and GFP (the cells stably expressed GFP to facilitate analysis of proliferation on CDM) are shown. Scale bar (actin) = 10 μm and scale bar (GFP) = 50 μm.**(g)** Western blot showing JMJD1a-GFP expression in FACS-sorted MDA-MB-231 cells based on the GFP intensity. GFP only was used as control. Non-paired t-test was used for all statistical analyses..

Supplementary figure 2

a

Patient nbr	Primary tumor location	Specimen site	Grade	TNM
Patient # 1	SCC linguae	Tongue	G2	T2N0M0
Patient # 2	Bucchal mucosa	Bucchal mucosa	G1	T2N2BM0
Patient # 3	SCC laryngis	Larynx	G2	T2N0M0

b

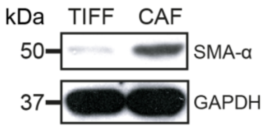

c

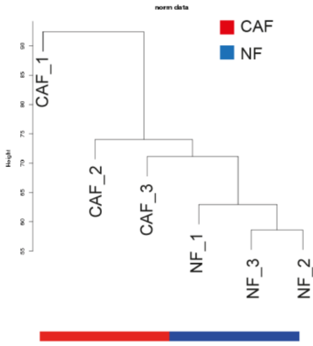

d

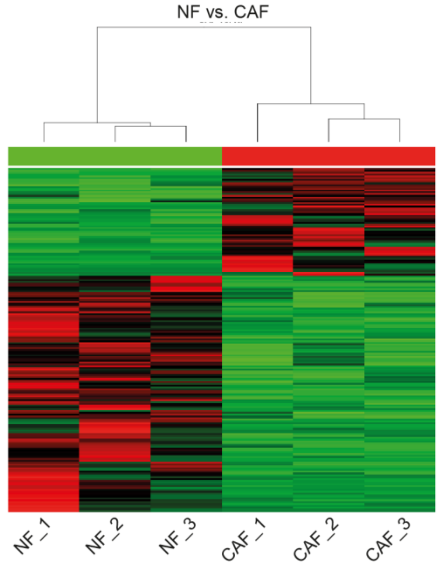

e

Most expressed genes in NFs and CAFs

ID	Gene
FN1	Fibronectin
VIM	Vimentin
COL1A1	Collagen 1a1
COL1A2	Collagen 1a2
GREM1	Gremlin 1
ACTB	Actin, beta
FLNA	Filamin A
ACTG1	Actin, gamma 1
PKM2	PKM2
MYH9	Myosin heavy chain 9
THBS1	Thrompospondin 1
KIAA1199	KIAA1199
MMP2	MMP2
SERPINE2	SERPINE2
FTL	Ferritin
COL3A1	Collagen 3 a1
COL6A1	Collagen 6 a 1
COL6A2	Collagen 6 a 2
COL6A3	Collagen 6 a 3
IGFBP5	Insulin-like growth factor binding prote
DCN	Decorin
CTSK	Cathepsin K
SPARC	SPARC
TGFBI	TGFBI
COL12A1	Collagen 12 a 1
IGFBP3	Insulin-like growth factor binding prote

f

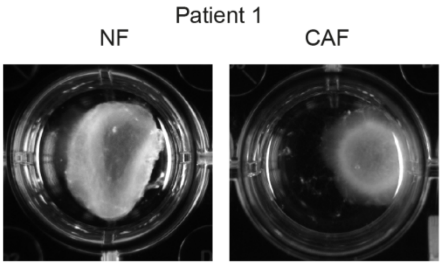

g

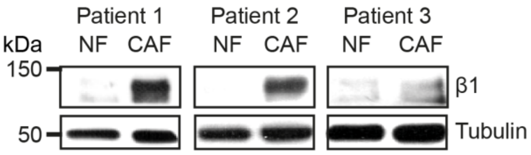

h

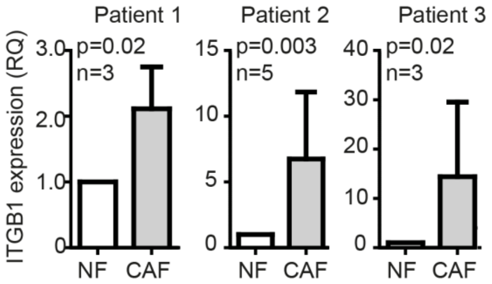

Supplementary Figure 2. Characterisation of the patient-derived NFs and CAFs.
(a) NFs from non-cancerous regions and CAFs from the tumour stroma were isolated from different regions of the head and neck of three patients. HNSCC cells were isolated from patient #2. **(b)** Representative western blot of α-SMA expression in TIFFs and patient #1 CAFs. **(c-d)** RNA expression profile-based clustering of the NFs and CAFs from three different SCC patients. Hierarchical clustering (c) and heat map blot (d) of the normalised RNA sequencing data. **(e)** A list of most commonly expressed genes according to RNA sequencing analysis in NFs and CAFs from three different SCC patients. **(f)** Collagen gel contraction assay. NFs and CAFs from patient #1 were embedded into type 1 collagen gels, and contraction was visualised after 3 days. **(g-h)** Representative western blot (g) and quantification (h) of β1-integrin expression in NFs and CAFs. The number of experiments (n) and p-values (paired t-test) are shown.

Supplementary figure 3

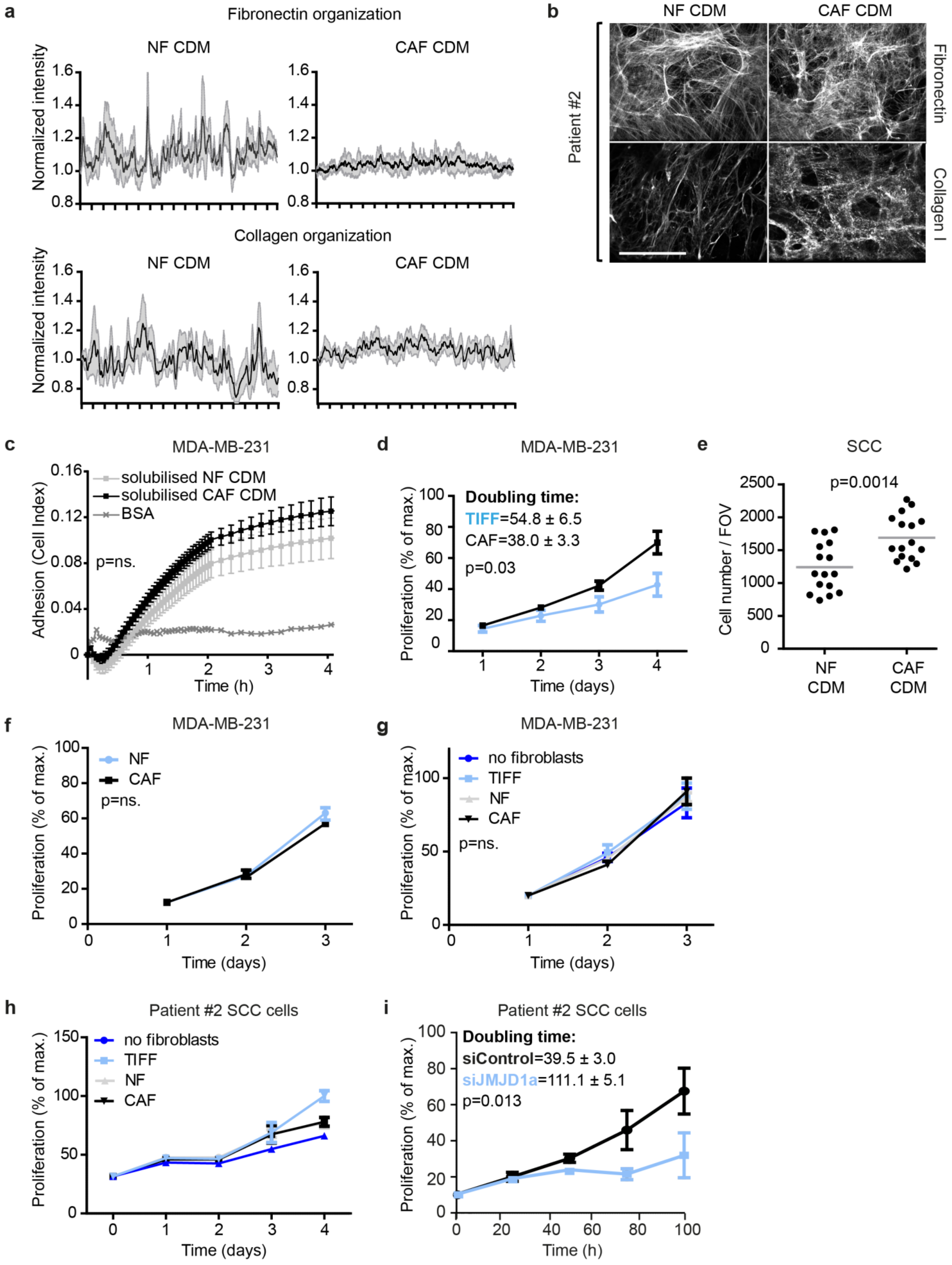

Supplementary Figure 3. Characterisation of the patient-derived NF and CAF matrices and their effects on cell proliferation.

(a) Line scans of collagen and fibronectin intensity across patient #1 NF and CAF CDMs. Black line=mean, gray area=s.d.. **(b)** Collagen I and fibronectin staining of patient #2 NF and CAF CDMs. **(c)** Adhesion of MDA-MB-231 cells to solubilised NF and CAF CDMs was measured in real-time. Adhesion to BSA was used as a control (mean±s.e.m.; n=4). **(d)** Proliferation of MDA-MB-231 on TIFF and patient #1-derived CAF CDMs over 4 days. The quantification of relative cell number over time is shown (mean±s.e.m.; n=3). **(e)** Proliferation of patient #2-derived SCC cells with NF or CAF CDM. Proliferation was analysed by scoring cell number in each field of view (FOV) with CellProfiler software after 4 days. (grey line=mean; n=16 images). **(f)** Proliferation of MDA-MB-231 cells in conditioned NF and CAF medium. Proliferation was analysed in Incucyte^{TM}-FLR (mean±s.e.m.; n=3). **(g-h)** Proliferation of MDA-MB-231 (f) and SCC cells (g) or in co-culture with patient #1 NFs or with patient #1 CAFs, TIFFs, or without fibroblasts. Cancer cells and fibroblasts were separated by filter and cancer cell proliferation was analysed daily with WST-1 staining. (mean±s.e.m.; n(MDA-MB-231)=3, n(SCC)=6. **(i)** Proliferation of patient-derived SCC cells upon JMJD1a silencing on plastic. Proliferation was analysed in Incucyte^{TM}-FLR. (mean±s.e.m.; n=3). Non-paired t-test was used for statistical analyses.

Supplementary Figure 4

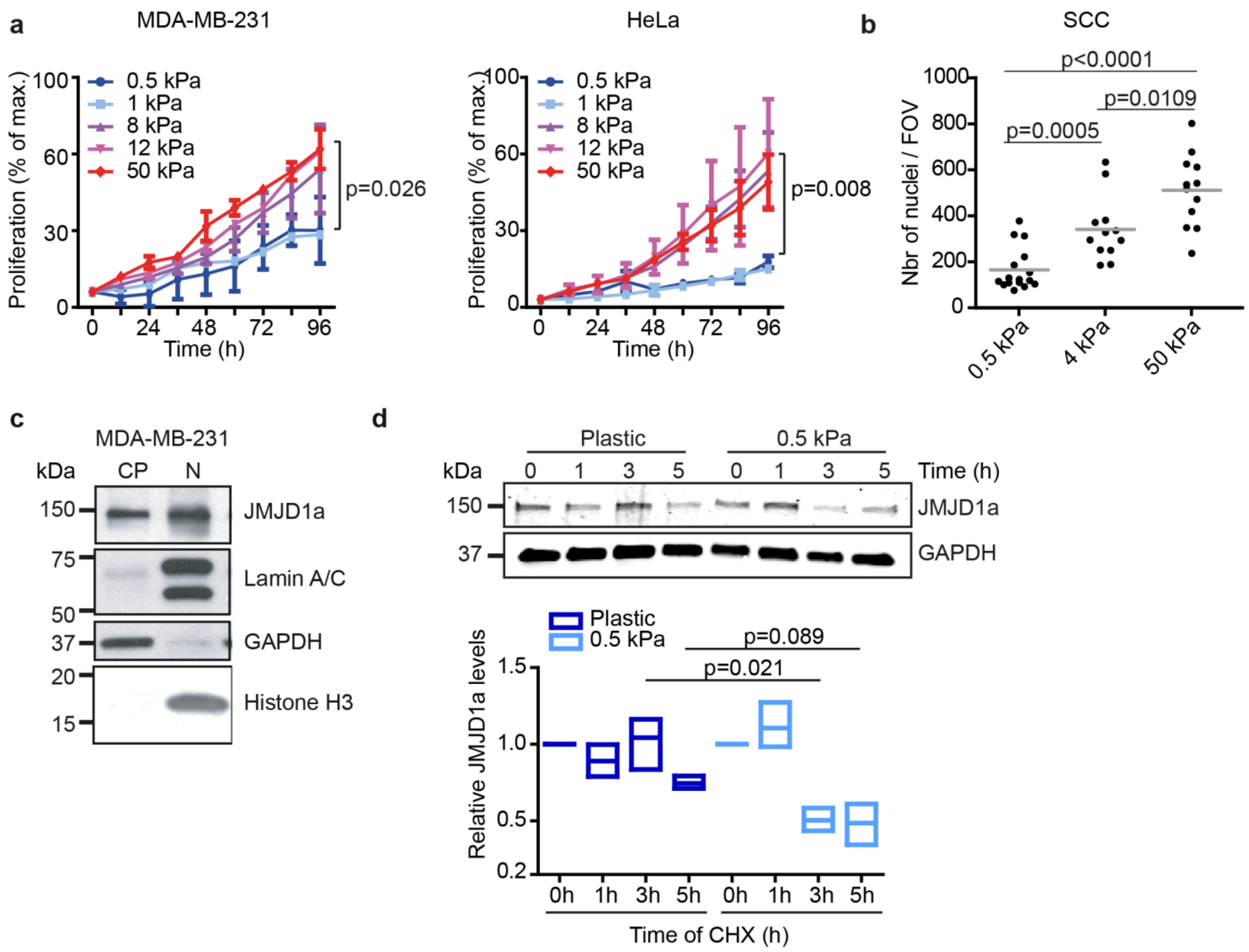

Supplementary Figure 4. Stiffness regulates cell proliferation.
(a) Proliferation of GFP-expressing MDA-MB-231 and HeLa cells on hydrogels of various stiffness. 5000 cells were plated on collagen I and fibronectin-coated hydrogels. Proliferation was analysed in Incucyte-FLR by analysing the cell number using GFP-expressing cells. P-value is calculates at last timepoint using non-paired t-test. **(b)** Proliferation of patient #2-derived SCC cells on hydrogels of various stiffness. 5000 cells were plated on hydrogels for 4 days. At day 4, cells were fixed and stained with dapi. Images were taken with 4 x objective and the number of nuclei in each field of view (FOV) was analysed with CellProfiler software. Non-paired t-test was used for statistical analysis. **(c)** Nuclear fractionation of MDA-MB-231 cells and western blot to analyse JMJD1a localisation. Lamin A/C and histone 3 were used as markers for nuclear fraction (N) and GAPDH for cytoplasmic fraction (CP). **(d)** Cycloheximide chase and western blot to analyse JMJD1a stability on plastic and on 0.5 kPa hydrogel. MDA-MB-231 cells were plated on collagen I and fibronectin coated hydrogels or on plastic for 24 h and cycloheximide (CXH) was added over indicated times. n=3. Paired t-test was used for statistical analysis.

Supplementary Figure 5

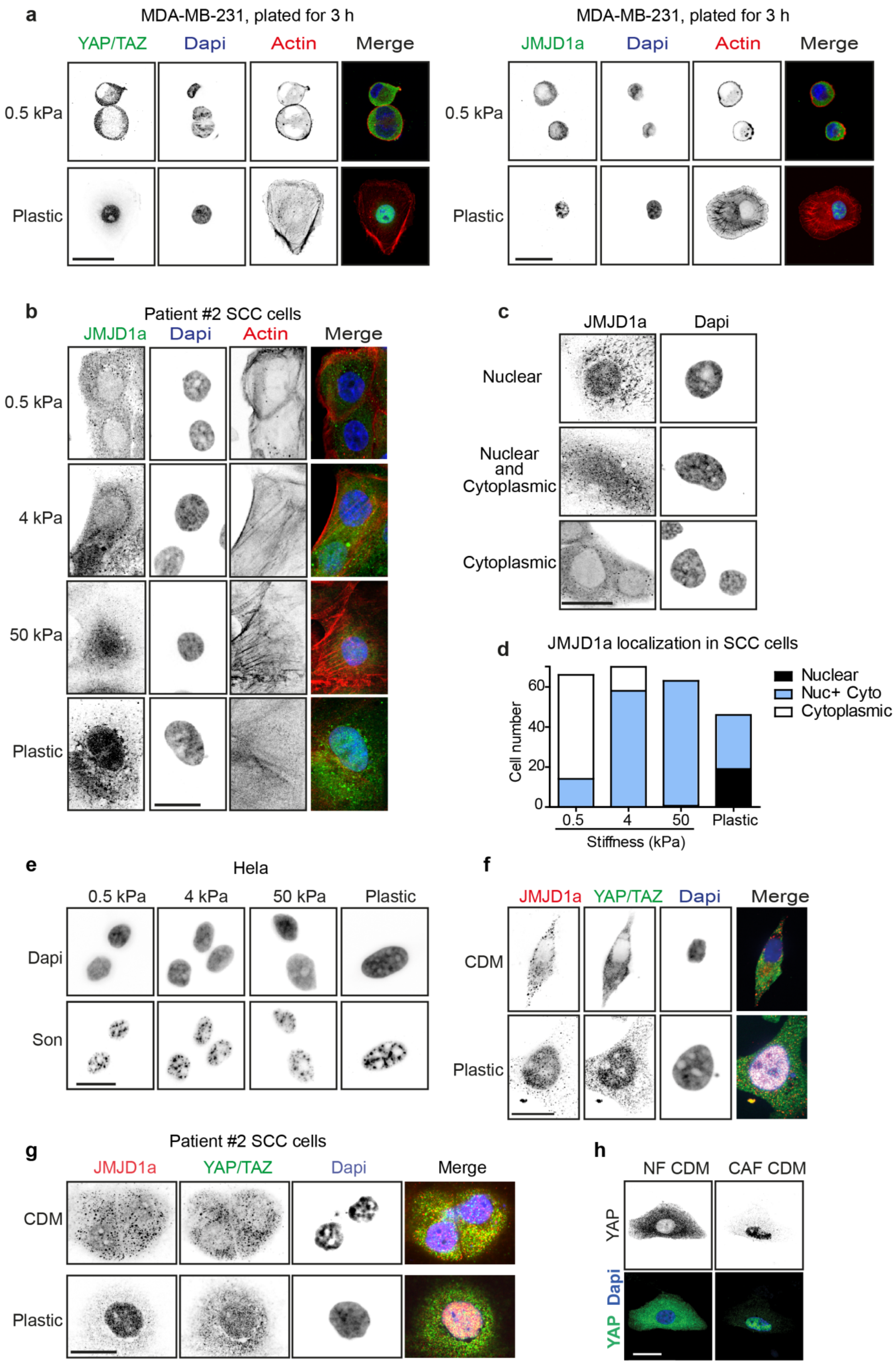

Supplementary Figure 5. Stiffness regulates JMJD1a localisation in SCC cells.
(a) Representative immunofluorescence staining of JMJD1a and YAP/TAZ after 3 h spreading on 0.5 kPa hydrogel or on plastic. Scale bar 20 µm. **(b)** JMJD1a staining in Patient#2 SCC cells cultured on hydrogels of varying stiffness over 3 days. Scale bar 10 µm. **(c)** Example images of cells highlighting differential localisation of JMJD1a (nuclear, nuclear and cytoplasmic and cytoplasmic only staining). Scale bar 10 µm. **(d)** Quantification of differential JMJD1a localisation (based on B) on 0.5 kPa, 4 kPa, 50 kPa hydrogels and on plastic. n=45-65 cells. **(e)** Immunofluorescence staining of a nuclear speckle marker Son in HeLa cells cultured on hydrogels of varying stiffness. Scale bar 10 µm. **(f)** Immunofluorescence staining showing JMJD1and YAP/TAZ localization in MDA-MB-231 cells of TIFF CDM and on plastic. Scale bar 10 µm. **(g)** JMJD1a (red) and YAP/TAZ (green) staining of patient #2 SCC cells on TIFF CDM after 3 days of culturing. Scale bar 10 µm. **(h)** Representative immunofluorescence staining showing YAP/TAZ localization in SCC cells on patient #2 derived NF and CAF CDM. Scale bar 10 µm.

Supplementary Figure 6

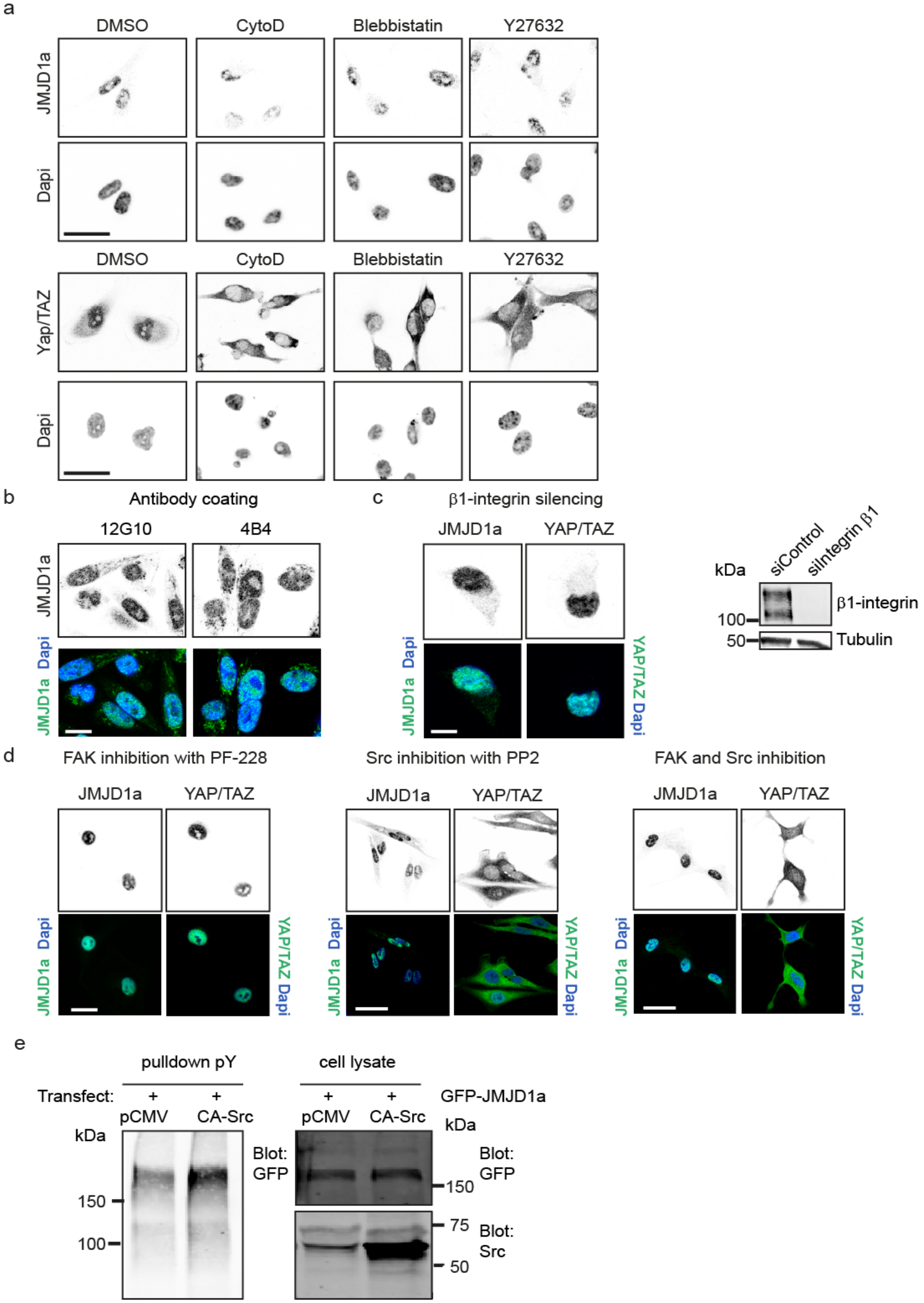

Supplementary figure 6. (a) Immunofluorescence staining showing JMJD1a and YAP/TAZ localization after 6 h treatment of DMSO-control, 1 μM Cytochalasin D (CytoD), 50 μM Blebbistatin and 50 μM ROCK inhibitor Y27632. Scale bar 20 μm. **(b)** MDA-MB-231 cells plated on integrin β1 antibody (12G10 or 4B4) coated plates over-night. Cells were fixed and stained with JMJD1a (green) and Dapi (blue). Scale bar 5 μm **(c)** Immunofluorescence staining of JMJD1a and YAP/TAZ in Integrin β1-silenced MDA-MB-231 cells plated on plastic. **(d)** MDA-MB-231 cells growing on plastic were treated with FAK inhibitor (PF-228, 10 μM), Src inhibitor (PP2, 10 μM) or in combination for 16 h. JMJD1a and YAP/TAZ localization was analysed by immunofluorescence staining. Scale bar 10 μm. (e) MDA-MB-231 cells were co-transfected with GFP-JMJD1a and pCMV (empty vector) or active-Src (CA-Src). Tyrosine phosphorylated proteins were isolated with anti-pY beads. Pull-downs and cell lysates were blotted as indicated. Shown is a representative experiment of two.

Supplementary figure 7

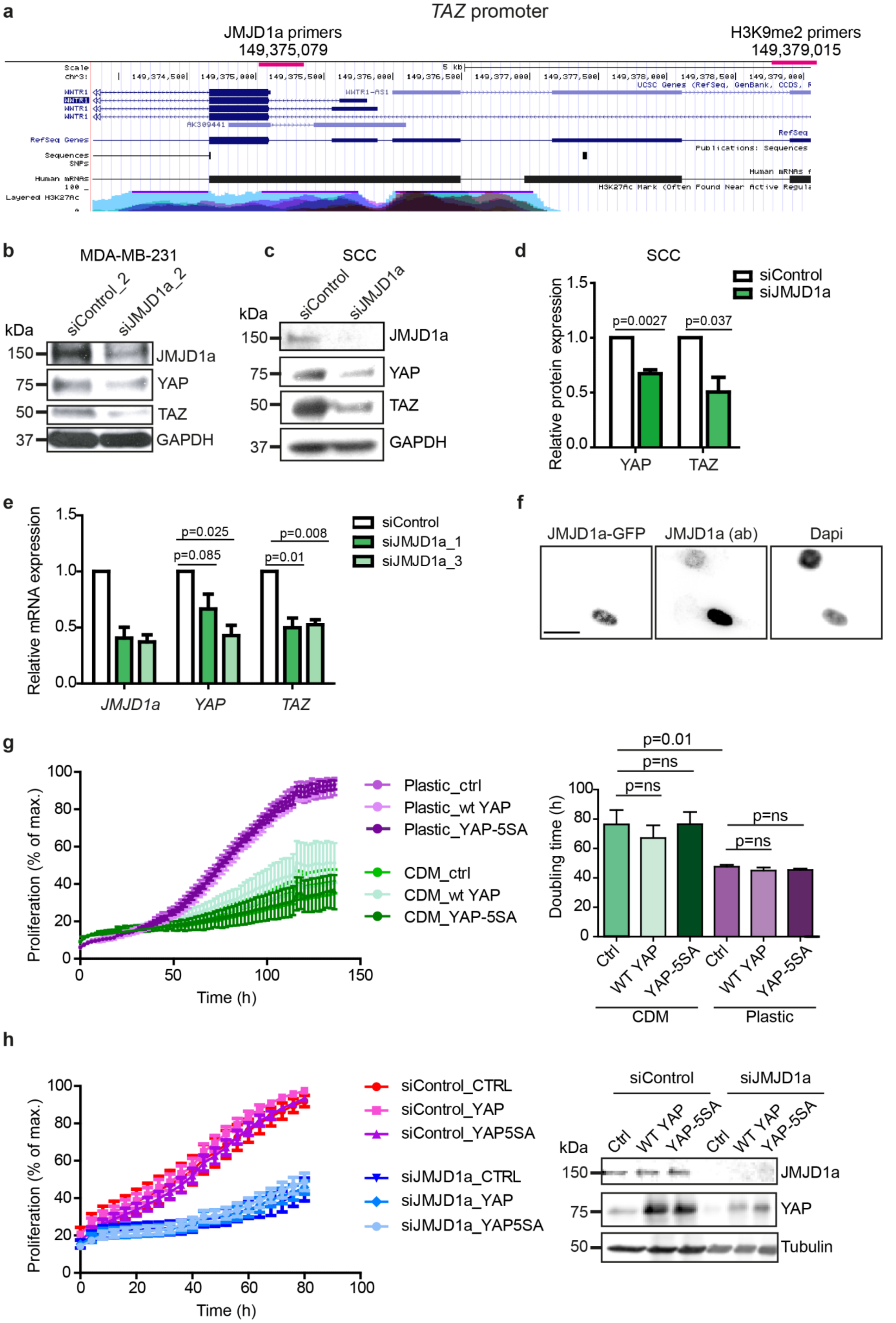

Supplementary Figure 7. JMJD1a regulates YAP/TAZ expression.

(a) Schematic representation of TAZ (WWTR1) promoter. Taken from UCSC Genome Browser on Human Feb. 2009 (GRCh37/hg19) Assembly. Genomic location of the qPCR which were used for JMJD1a and H3K9me2 ChIP are indicated in red. **(b)** Representative western blot showing YAP/TAZ expression in MDA-MB-231 upon JMJD1a silencing using a second independent siRNA. **(c-d)** Western blot (b) and quantification (c) of YAP/TAZ expression upon JMJD1a silencing in patient#2-derived SCC cells. Data are mean ±s.d. and n=4 **(e)** Taqman qRT-PCR of JMJD1a (*KDM3A*), *YAP* and *TAZ* mRNA levels in patient #2-derived SCC cells transfected with control siRNA or two independent *JMJD1a*-targeting oligos (siJMJD1a #1 and #3). Data are mean±s.d. and n=3. **(f)** A representative immunofluorescence image of a JMJD1a-GFP overexpressing MDA-MB-231 cell. JMJD1a overexpression was additionally confirmed using a JMJD1a-specific antibody (ab). Scale bar 20 µm. **(g)** Proliferation of MDA-MB-231 cells on CDM and plastic. Cells were transfected with control plasmid, wt-YAP or YAP-5SA mutant and proliferation was monitored in Incucyte-ZOOM live cell incubator. Data are mean±s.e.m. and n=4 for each condition. **(h)** MDA-MB-231 cells were transfected with control or JMJD1a siRNA for 48 h followed by transfection with control, wt-YAP or YAP-5SA plasmids. Data are mean±s.e.m. and n=4 for each condition. Paired t-test was used for statistical analyses in.

Supplementary Figure 8

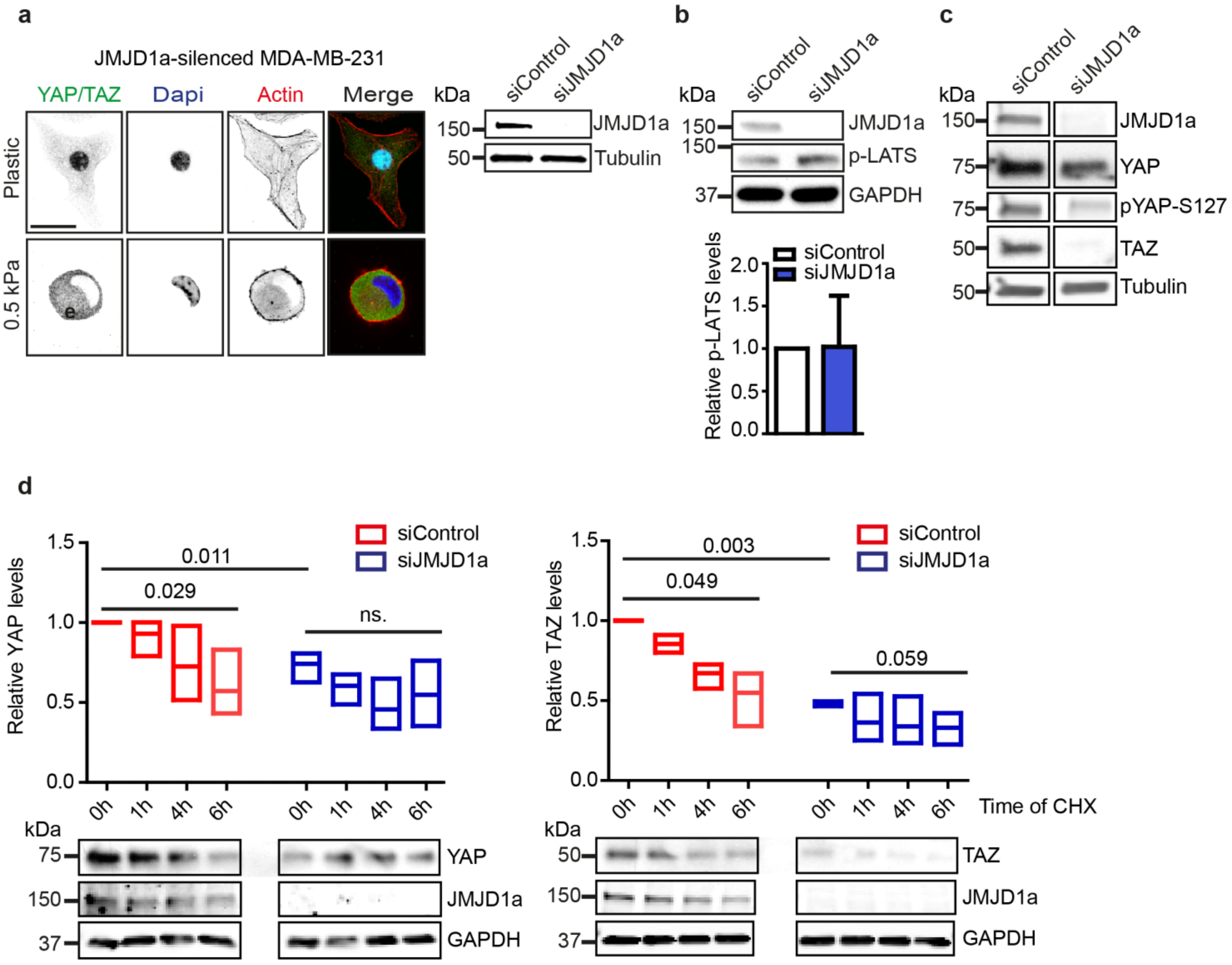

Supplementary Figure 8. (a) Immunofluorescence staining of YAP/TAZ on 0.5 kPa hydrogels and on plastic in JMJD1a-silenced MDA-MB-231 cells. Western blot shows the JMJD1a silencing efficiency. Scale bar 20 µm. **(b)** Representative western blot and quantification showing pLATS levels in JMJD1a-silenced MDA-MB-231 cells. (mean±s.d.; n=4). **(c)** Representative western blot showing YAP/TAZ and phosphorylated YAP (S127) levels in JMJD1a-silenced MDA-MB-231 cells. **(d)** Quantification and representative western blot showing YAP/TAZ stability in control and JMJD1a siRNA transfected cells. Protein stability was studied by cycloheximide (CHX) treatment over indicated time points. n=3.Line at mean is shown ±s.d. Paired t-test was used for statistical analysis.

Supplementary Figure 9

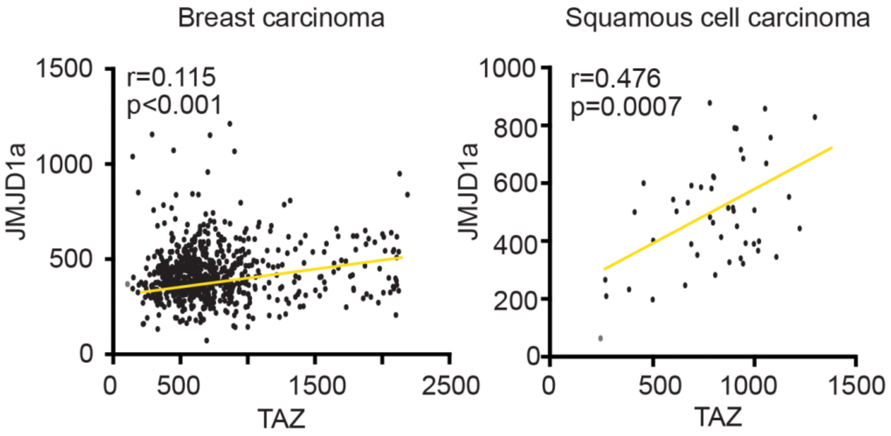

Supplementary Figure 9. JMJD1a and YAP levels correlate in human cancer.
Correlation of JMJD1a and TAZ expression in breast carcinoma and squamous cell carcinoma (SCC) patients. n (breast carcinoma)=720 and n(SCC)=46. Linear trend line is shown in yellow. Non-parametric Mann-Whitney was used for statistical analyses.

Supplementary figure 10. Uncropped scans of the most important blots

Figure 2a

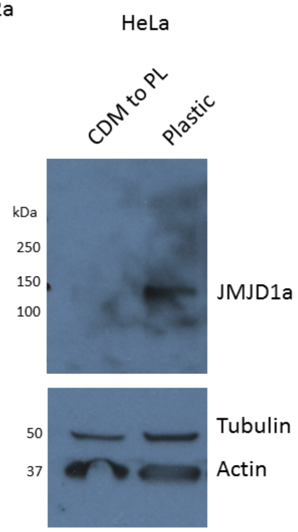

Figure 2b

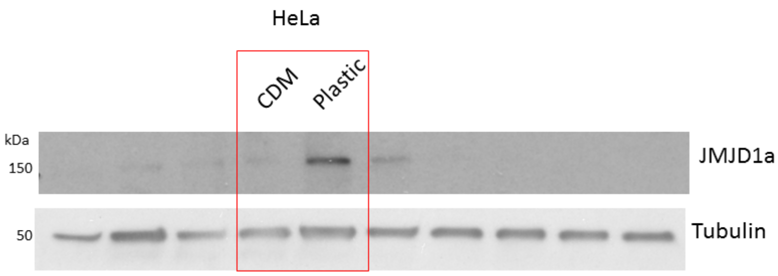

Figure 3h

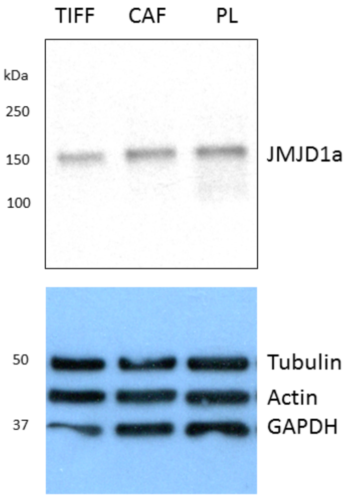

Figure 5g

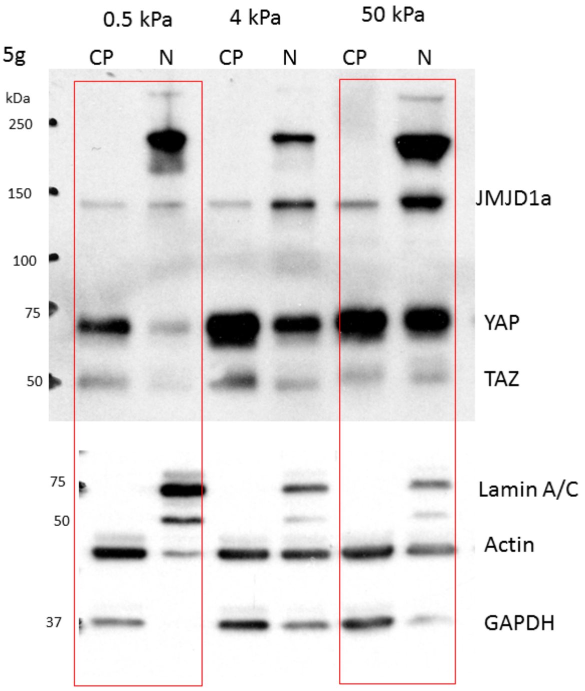

Figure 6n

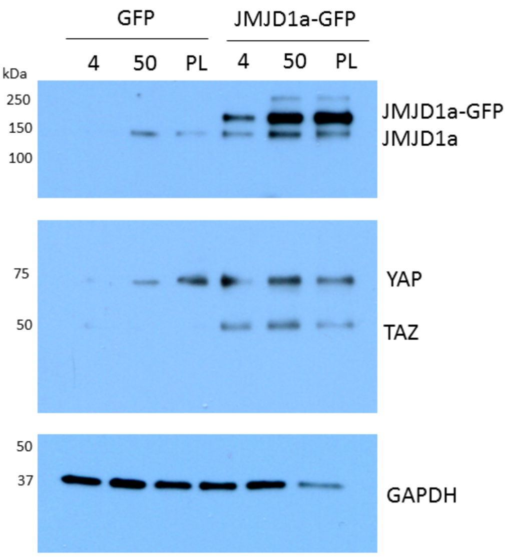

Supplementary Figure 8d

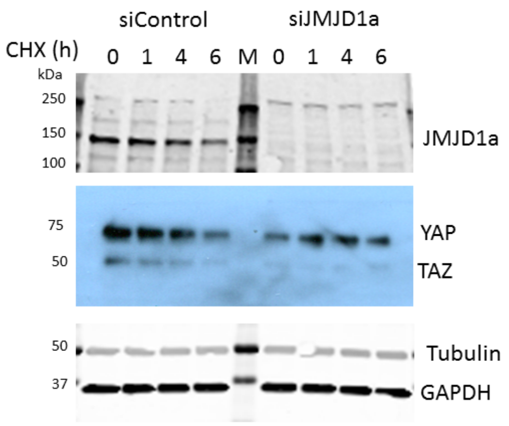

M= Molecular marker

Figure 6m

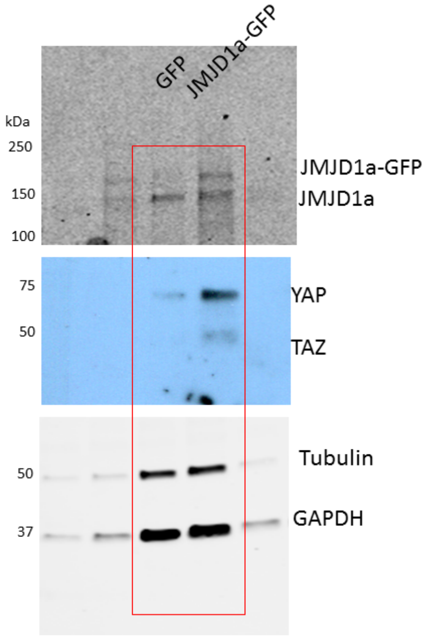

Supplementary Note 1

(Related to supplementary tables 1-4). JMJD1a and YAP/TAZ expression the primary breast tumors of the patients entered to a randomized prospective trial (the FinHer trial, Joensuu H et al. NEJM 2006) were strongly associated with several commonly assessed clinicopathological prognostic factors. We stained a breast carcinoma tissue-microarray consisting of breast tumor samples from 746 (73.9%) of the 1,010 patients entered to the FinHer trial for JMJD1a and YAP/TAZ (the YAP antibody recognizes both transcription factors). 689 (94.3%) and 262 (35.8%) out of the 731 tumors available for JMJD1a staining had positive cytoplasmic and nuclear staining, respectively, and 645 (86.5%) and 514 (68.9%) out of the 746 cancers available for YAP/TAZ staining had positive cytoplasmic and nuclear YAP/TAZ expression. Cytoplasmic JMJD1a expression was significantly (P <0.05) associated with negative estrogen receptor (ER) expression and progesterone receptor (PgR) expression (each assessed by immunohistochemistry), amplification of *HER2* (assessed with chromogenic in situ hybridization), and the ductal histological type, whereas nuclear JMJD1a expression was significantly associated with *HER2* amplification and p53 expression, but not with ER or PgR expression. Neither cytoplasmic nor nuclear JMJD1a expression was associated with cancer histological grade or primary tumor size (Supplementary tables x and x). Both cytoplasmic and nuclear YAZ/TAZ expression had very strong (P <0.0001) associations with negative tumor

ER expression and the triple negative (ER-, PgR-, *HER2*-) phenotype, and they were significantly associated also with negative PgR expression, poor histological grade of differentiation, and p53 expression (Supplementary tables x and x). JMJD1a expression was not associated with presence of axillary nodal metastases, YAP/TAZ expression was associated with absence of axillary nodal metastases, and neither cytoplasmic nor nuclear expression of JMJD1a or YAP/TAZ was significantly associated with distant disease free-survival or overall survival in the FinHer series (P >0.10 for each comparison).

Supplementary Table 1

Association with JMJD1A cytoplasmic expression with clinicopathological parameters, when JMJD1A1 categories 0=negative, 1=low, 2=medium-high

Parameter	JMJD1A			P (chi-squared)
	0	1	2	
ER				
Positive	34 (6.7)	248 (48.7)	227 (44.6)	
Negative	8 (3.6)	94 (42.3)	120 (54.1)	0.033
PgR				
Positive	31 (7.5)	196 (47.3)	187 (45.2)	
Negative	11 (3.5)	146 (46.1)	160 (50.5)	0.046
HER2				
Positive	2 (1.2)	77 (44.5)	94 (54.3)	
Negative	40 (7.2)	265 (47.5)	253 (45.3)	0.005
Biological Group				
ER+, HER2-	35 (7.9)	212 (48.1)	194 (44.0)	
ER+, HER2+	0 (0)	40 (50.0)	40 (50.0)	
ER-, HER2+	2 (2.2)	35 (38.5)	54 (59.3)	
ER-, HER2-	5 (4.2)	55 (46.2)	59 (49.6)	N.C.*
Histological grade				
Gr. 1	4 (4.4)	44 (48.9)	42 (46.7)	
Gr. 2	22 (7.5)	135 (45.9)	137 (46.6)	
Gr. 3	14 (4.4)	150 (47.5)	152 (48.1)	0.556
P53 expression				
No	34 (6.5)	243 (46.6)	245 (46.9)	
Yes	7 (3.7)	87 (46.3)	94 (50.0)	0.344

Tumor histology				
Ductal	29 (5.2)	261 (46.7)	296 (48.1)	
Other	13 (9.0)	81 (55.9)	51 (35.2)	0.011
pT				
1	19 (6.3)	132 (44.0)	149 (49.7)	
2	19 (5.1)	179 (48.4)	172 (46.5)	
3	3 (6.8)	22 (50.0)	19 (43.2)	
4	1 (5.9)	9 (52.9)	7 (41.2)	0.907
pN				
0	4 (5.6)	38 (48.1)	37 (46.8)	
1	36 (5.7)	292 (46.5)	300 (47.8)	
2 or 3	2 (8.7)	12 (52.2)	9 (39.1)	0.917

*Not calculated due to small cell frequencies in JMJD1A category 0

Supplementary Table 2
Association with JMJD1A nuclear expression with clinicopathological parameters

Parameter	JMJD1A nuclear expression		P (chi-squared)
	negative	positive	
ER			
Positive	323 (63.6)	185 (36.4)	
Negative	146 (65.5)	77 (34.5)	0.624
PgR			
Positive	264 (63.8)	150 (36.2)	
Negative	205 (64.7)	112 (35.3)	0.801
HER2			
Positive	96 (55.5)	77 (44.5)	
Negative	373 (66.8)	185 (33.2)	0.007
Biological Group			
ER+, HER2-	288 (65.3)	153 (34.7)	
ER+, HER2+	43 (53.8)	37 (46.3)	
ER-, HER2+	51 (56.0)	40 (44.0)	
ER-, HER2-	87 (73.1)	32 (26.9)	0.013
Histological grade			
Gr. 1	62 (68.9)	28 (31.1)	
Gr. 2	190 (64.6)	104 (35.4)	
Gr. 3	195 (61.7)	121 (38.3)	0.429
P53 expression			
No	348 (66.7)	174 (33.3)	
Yes	107 (56.9)	81 (43.1)	0.017
Tumor histology			
Ductal	370 (63.1)	216 (36.9)	
Other	99 (68.3)	46 (31.7)	0.248

pT			
1	191 (63.7)	109 (36.3)	
2	239 (64.6)	131 (35.4)	
3 or 4	39 (63.9)	22 (36.1)	0.969
pN			
0	57 (72.2)	22 (27.8)	
1, 2 or 3	411 (63.1)	240 (36.9)	0.115

*Mann-Whitney test

Supplementary Table 3

Association with YAP/TAZ cytoplasmic expression with clinicopathological parameters, when YAP/TAZ categories 0=negative, 1=low, 2=medium, 3=high

Parameter	YAP/TAZ cytoplasmic expression				P (chi-squared)
	0	1	2	3	
ER					
Positive	73 (14.0)	273 (52.3)	124 (23.8)	52 (10.0)	
Negative	28 (12.5)	72 (32.1)	60 (26.8)	64 (28.6)	<0.0001
PgR					
Positive	51 (12.2)	219 (52.4)	104 (24.9)	44 (10.5)	
Negative	50 (15.3)	125 (38.2)	80 (24.5)	72 (22.0)	<0.0001
HER2					
Positive	28 (15.9)	70 (39.8)	56 (31.8)	22 (12.5)	
Negative	73 (12.8)	275 (48.2)	128 (22.5)	94 (16.5)	0.027
Biological Group					
ER+, HER2-	61 (13.5)	237 (52.6)	105 (23.3)	48 (10.6)	
ER+, HER2+	15 (18.3)	37 (45.1)	24 (29.3)	6 (7.3)	
ER-, HER2+	13 (14.1)	32 (34.8)	32 (34.8)	15(16.3)	
ER-, HER2-	12 (9.9)	39 (32.2)	23 (19.0)	47 (38.8)	<0.0001
Histological grade					
Gr. 1	5 (5.4)	51 (54.8)	27 (29.0)	10 (10.8)	
Gr. 2	40 (13.4)	152 (50.8)	71 (23.7)	36 (12.0)	
Gr. 3	53 (16.5)	123 (38.2)	82 (25.5)	64 (19.9)	0.001
P53 expression					
No	80 (14.7)	269 (49.5)	124 (22.8)	70 (12.9)	

Yes	21 (11.1)	67 (35.3)	57 (30.0)	45 (23.7)	0.0001
Tumor histology					
Ductal	80 (13.3)	269 (44.8)	154 (25.6)	98 (16.3)	
Other	21 (14.5)	76 (52.4)	30 (20.7)	18 (12.4)	0.274
pT					
1	32 (10.3)	143 (46.0)	93 (29.9)	43 (13.8)	
2	56 (15.0)	175 (46.8)	80 (21.4)	63 (16.8)	
3 or 4	13 (21.3)	27 (44.3)	11 (18.0)	10 (16.4)	0.043
pN					
0	17 (21.3)	31 (38.8)	13 (16.3)	19 (23.8)	
1, 2 or 3	84 (12.6)	314 (47.3)	170 (25.6)	97 (14.6)	0.010

Supplementary Table 4

Association with YAP/TAZ nuclear expression with clinicopathological parameters

Parameter	YAP/TAZ nuclear expression		P (chi-squared)
	0	1	
ER			
Positive	393 (75.3)	129 (24.7)	
Negative	121 (54.0)	103 (46.0)	<0.0001
PgR			
Positive	303 (72.5)	115 (27.5)	
Negative	210 (64.2)	117 (35.8)	0.016
HER2			
Positive	121 (68.8)	55 (31.3)	
Negative	393 (68.9)	177 (31.1)	0.961
Biological Group			
ER+, HER2-	338 (74.9)	113 (25.1)	
ER+, HER2+	61 (74.4)	21 (25.6)	
ER-, HER2+	59 (64.1)	33 (35.9)	
ER-, HER2-	56 (46.3)	65 (53.7)	<0.0001
Histological grade			
Gr. 1	66 (71.0)	27 (29.0)	
Gr. 2	228 (76.3)	71 (23.7)	
Gr. 3	199 (61.8)	123 (38.2)	0.0005
P53 expression			
No	390 (71.8)	153 (28.2)	
Yes	116 (61.1)	74 (38.9)	0.006

Tumor histology			
Ductal	409 (68.1)	192 (31.9)	
Lobular	105 (72.4)	40 (27.6)	0.309
pT			
1	220 (70.7)	91 (29.3)	
2	251 (67.1)	123 (32.9)	
3 or 4	43 (70.5)	18 (29.5)	0.571
pN			
0	41 (51.3)	39 (48.8)	
1, 2 or 3	472 (71.0)	193 (29.0)	0.0003

Supplementary table 5

The qPCR primers and universal probe library (UPL) probes used in the study.

Gene	Forward primer	Reverse primer	UPL probe nbr
JMJD1a	CCAGCCTCAAAGGAAGACCT	ACTGCACCAAGAGTGGTTT	71
YAP	TGGATTTTGAGTCCCACCAT	ATCCCAGCACAGCAAATTCT	48
TAZ	ATTCGAATGCGCCAAGAG	AACTGGGGCAAGAGTCTCAG	4
CTGF	CTCCTGCAGGCTAGAGAAGC	GATGCACTTTTTGCCCTTCTT	56
THBS1	TGGAGACCAGCCATCGTC	CAATGCCACAGTTCCTGATG	85
GAPDH	GCCCAATACGACCAAATCC	AGCCACATCGCTCAGACA	60
